# Nuclear moonlighting of cytosolic glyceraldehyde-3-phosphate dehydrogenase regulates Arabidopsis response to heat stress

**DOI:** 10.1038/s41467-020-17311-4

**Published:** 2020-07-10

**Authors:** Sang-Chul Kim, Liang Guo, Xuemin Wang

**Affiliations:** 1 Department of Biology, University of Missouri-St. Louis, St. Louis, MO 63121 USA; 2 Donald Danforth Plant Science Center, St. Louis, MO 63132 USA; 3 Present Address: National Key Laboratory of Crop Genetic and Improvement, College of Plant Science and Technology, Huazhong Agricultural University, Wuhan, 430070 China

**Keywords:** Molecular biology, Molecular biology, Plant sciences, Plant sciences

## Abstract

Various stress conditions induce the nuclear translocation of cytosolic glyceraldehyde-3-phosphate dehydrogenase (GAPC), but its nuclear function in plant stress responses remains elusive. Here we show that GAPC interacts with a transcription factor to promote the expression of heat-inducible genes and heat tolerance in Arabidopsis. GAPC accumulates in the nucleus under heat stress. Overexpression of *GAPC* enhances heat tolerance of seedlings and the expression of heat-inducible genes whereas knockout of *GAPCs* has opposite effects. Screening of Arabidopsis transcription factors identifies nuclear factor Y subunit C10 (NF-YC10) as a GAPC-binding protein. The effects of *GAPC* overexpression are abolished when *NF-YC10* is deficient, the heat-induced nuclear accumulation of GAPC is suppressed, or the GAPC-NF-YC10 interaction is disrupted. *GAPC* overexpression also enhances the binding ability of NF-YC10 to its target promoter. The results reveal a cellular and molecular mechanism for the nuclear moonlighting of a glycolytic enzyme in plant response to environmental changes.

## Abstract (web summary)

Stress conditions can induce translocation of glyceraldehyde-3-phosphate dehydrogenase (GAPC) to the nucleus. Here Kim et al. show that in Arabidopsis, GAPC can interact with the NF-YC transcription factor subunit, enhance expression of heat-inducible genes and promote heat tolerance.

## Introduction

Glyceraldehyde-3-phosphate dehydrogenase (GAPDH) is a glycolytic enzyme converting glyceraldehyde-3-phopshate to 1,3-bisphosphoglycerate. Glycolysis breaks down carbohydrates, providing intermediates for energy production and cellular metabolism, including the synthesis of fatty acids, amino acids, hormones, and osmolytes for drought and salinity protection. Arabidopsis has eight members of GAPDH, two of which are cytosolic forms GAPC1 and GAPC2^1,2^. Increasing evidence suggests that GAPC also plays important roles in mediating plant response to abiotic and biotic stresses^3,4^. For example, GAPC is involved in plant response to oxidative stress that could result from abiotic and biotic challenges^5^. GAPC has been implicated in plant response to stresses, including cadmium, long-chain bases (dihydrosphingosine), reactive oxygen species, the lipid mediator phosphatidic acid (PA), and cold-induced sweetening and apical dominance of potato^6–10^. We showed that GAPC was involved in mediating plant water loss and Arabidopsis mutants deficient in *GAPC1* and *GAPC2* suffered a higher transpirational water loss than wild type plants^2^. GAPC affected multiple plant immune responses to bacterial pathogen, such as reactive oxygen species production, programmed cell death, and autophagy^8,11^. GAPC was also involved in viral infection^10,12^.

One mechanism for the GAPC’s action in stress response is its stress-induced nuclear translocation. A small pool of GAPC accumulated in the nucleus in Arabidopsis response to treatments with cadmium, bacterial flagellin, PA, and hydrogen sulfide^6–8,13,14^. The nuclear accumulation of GAPC was also observed in tobacco BY-2 (bright-yellow 2) cells exposed to long-chain bases, regulators for programmed cell death in plants^15^. Since GAPC has no nuclear localization signal, post-translational modifications of specific amino acid residues are believed to be important for the stress-induced intracellular translocation. Under certain stress conditions, the highly reactive catalytic cysteine of GAPC undergoes thiol modifications, such as *S*-nitrosylation, *S*-sulfhydration, and *S*-glutathionylation^13,14^ whereas specific lysines can be acetylated to promote nuclear translocation^16^. In addition, lysine ubiquitination by an E3 ubiquitin-ligase was reported as a potential mechanism for GAPC nuclear localization^17^.

Even though some of the mechanisms required for stress-induced nuclear localization is now determined, a clear role of nuclear GAPC remains elusive in plant stress responses. In animal cells, nuclear GAPDH affects transcriptional activity, DNA replication and repair, and epigenetic modifications by binding to various nuclear components, including transcriptional machinery and nucleic acids^18,19^. Because of its potential implication in diverse cellular phenomena, GAPDH is currently being investigated in context with human disorders, including neurodegenerative diseases (e.g. Alzheimer’s disease) and cancer^20–23^. In plants, one report indicated that GAPC directly interacted with a DNA sequence encoding NADP-dependent malate dehydrogenase, but the effect of such binding is unknown^13^. Rice GAPC1 was found to bind to the promoters of some glycolytic genes including *GAPC1* itself, indicating that GAPC is a transcriptional activator of glycolytic function^16^. This study was undertaken to determine how GAPC affected nuclear function in plant stress responses. Here we show that GAPC interacts with the transcription factor nuclear factor Y subunit C10 (NF-YC10) and regulates transcriptional and physiological responses to heat stress.

## Results

### NF-YC10 is identified as a GAPC-binding transcription factor

**Fig. 1:**
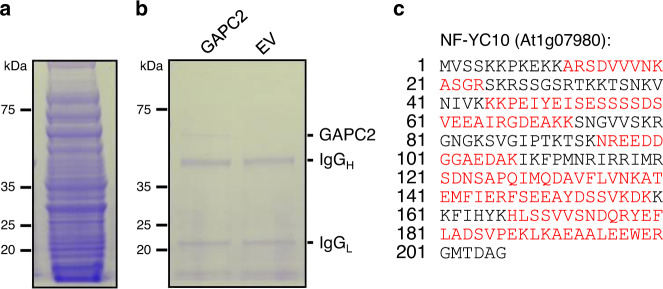
Screening of Arabidopsis transcription factors to identify GAPC-binding proteins. **a** Mixture of the purified transcription factors. Proteins were purified by affinity chromatography and separated on a polyacrylamide gel. The gel was stained with Coomassie Brilliant Blue. Protein marker size is on the left. **b** Gel image of co-immunoprecipitation. Transcription factors co-immunoprecipitated with GAPC2-Flag or empty vector control (EV) were separated on a polyacrylamide gel and stained with Coomassie Brilliant Blue. Protein marker size is on the left. Positions of GAPC2, immunoglobulin G (IgG) heavy and light chains are on the right. **c** Mass spectrometry-based identification of NF-YC10. Trypsin-digested peptides from the immunoprecipitated samples were sequenced by LC-MS/MS. Shown here is NF-YC10 sequence with the unique peptides in red that were identified with probability >99%.

The nuclear translocation of GAPC under stress raises a possibility that GAPC may play a role in stress-responsive gene expression by modulating transcriptional activity of transcription factor(s) through direct protein-protein interaction. To test this possibility, we screened an Arabidopsis transcription factor library for transcription factors potentially binding to and modulated by GAPC. We modified an Arabidopsis cDNA library composed of ~1500 transcription factors^24^ to produce recombinant proteins in *Escherichia coli*. After a library-efficiency transformation, colonies were pooled together, from which the recombinant proteins were isolated as a mixture. A large number of different proteins expressed in the *E. coli* clones was verified by separation of the proteins on a polyacrylamide gel (Fig. 1a). The protein mixture was then co-immunoprecipitated with GAPC2-Flag that was purified from Arabidopsis overexpressing the recombinant protein or from control plants with empty vector (EV) using an anti-Flag antibody. SDS-PAGE analysis revealed the successful immunoprecipitation of GAPC2 as determined by the clear GAPC2 band (and immunoglobulin G heavy/light chain bands; Fig. 1b). To identify proteins co-immunoprecipitated with GAPC2, we sequenced the entire immunoprecipitants by mass spectrometry and compared the identified proteins between the GAPC2 sample and EV control. The mass spectrometry-based protein sequencing identified the nuclear factor Y subunit C10 (NF-YC10) co-precipitated specifically with GAPC2 (Fig. 1c).

### GAPC interaction with NF-YC10 occurs in vitro and in planta

**Fig. 2:**
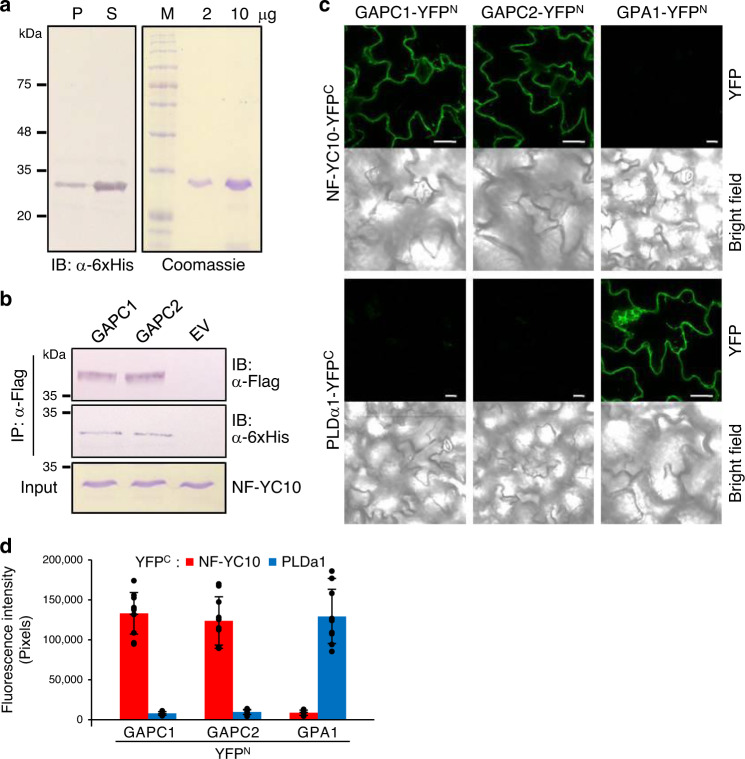
Physical interaction of GAPC with NF-YC10. **a** Expression and purification of recombinant NF-YC10 produced in *E. coli*. Proteins from pellet (P) or supernatant (S) following centrifugation of *E. coli* cell lysate were separated, and NF-YC10-6xHis was probed by immunoblotting (IB) with an anti-6xHis antibody (left panel). Protein marker size is on the left. NF-YC10 was nickel affinity-purified from the *E. coli* and shown here on polyacrylamide gel stained with Coomassie blue (right panel). M, marker. **b** Co-immunoprecipitation of GAPC and NF-YC10. GAPC1-Flag, GAPC2-Flag, or empty vector control (EV) was mixed with NF-YC10-6xHis. Immunoprecipitation (IP) was performed using an anti-Flag antibody, then immunoblotting (IB) was performed using the antibodies indicated on the right. NF-YC10 protein input is shown on polyacrylamide gel stained with Coomassie blue (bottom panel). **c** Bimolecular fluorescence complementation (BiFC) analysis. GAPC-YFP^N^ and NF-YC10-YFP^C^ were transiently co-expressed in tobacco leaves and observed under a confocal microscope. GPA1 and PLDα1 that bound each other were used as controls. Scale bars = 10 µm. **d** Quantification of BiFC. Fluorescence intensity of tobacco leaves used in **c** was measured by ImageJ software. Values are average ± S.D. from 10 randomly chosen regions of tobacco leaves infiltrated. Black dots represent individual data points.

To verify the interaction between GAPC and NF-YC10, we cloned *NF-YC10* from Arabidopsis and expressed the recombinant protein as a fusion with 6xHis tag in *E. coli*. The recombinant NF-YC10 was present mostly in the soluble fraction of *E. coli* cell lysate and purified to near homogeneity (Fig. 2a). The purified NF-YC10 was then co-immunoprecipitated using an anti-Flag antibody with GAPC1-Flag or GAPC2-Flag purified from Arabidopsis overexpressing the respective proteins or proteins purified from control plants with empty vector (EV). Immunoblotting analysis using an anti-6xHis antibody demonstrated that NF-YC10 was co-precipitated with both GAPC1 and GAPC2, but not with EV control (Fig. 2b). Next, we performed a bimolecular fluorescence complementation (BiFC) assay to verify the GAPC-NF-YC10 interaction in planta. GAPC1 or GAPC2 fused with the N-terminal half of yellow fluorescence protein (GAPC-YFP^N^) and NF-YC10 with C-terminal half of YFP (NF-YC10-YFP^C^) were co-expressed transiently in tobacco leaves, and the fluorescent signal was observed under a confocal microscope. For both GAPC1 and GAPC2, co-expression of GAPC and NF-YC10 produced detectable fluorescence in the tobacco leaves, but the signal was not observed when GAPC was replaced by a non-NF-YC10-binding protein the Gα subunit of heterotrimeric protein (GPA1; Fig. 2c). Likewise, no fluorescent signal was detected when NF-YC10 was replaced by phospholipase Dα1 (PLDα1; Fig. 2c). As GPA1 and PLDα1 interact with each other^25,26^, fluorescence was detected upon their co-expression (Fig. 2c). The fluorescent signal from co-expression of GAPC and NF-YC10 was quantified to be markedly greater than when a counterpart was replaced by GPA1 or PLDα1 (Fig. 2d). Taken together, these results indicate that the GAPC-NF-YC10 interaction occurs both in vitro and in vivo.

### GAPC modulates heat tolerance in Arabidopsis

**Fig. 3:**
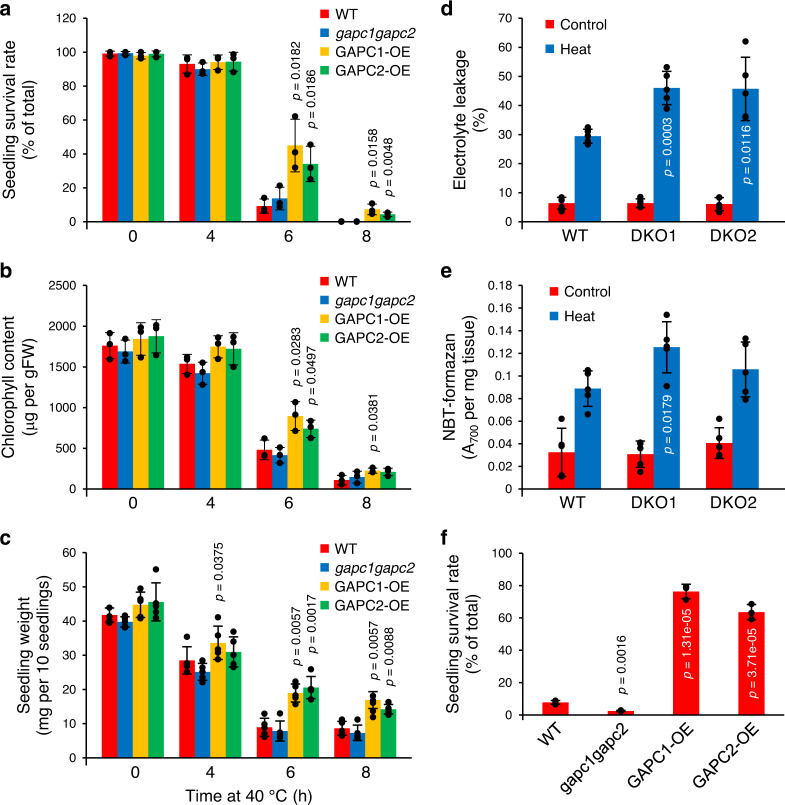
Phenotypic response of *GAPC*-altered Arabidopsis to heat. **a–c** Quantitative measurements of growth response to heat. Five-day-old seedlings were treated at 40 °C for the indicated times, then placed back to normal temperature condition. Plants were measured for seedling survival rate (**a**), chlorophyll content (**b**), and seedling weight (**c**). Values are average ± S.D. *P* values indicate significant difference from WT determined by two-tailed student’s *t* test (*n* = 3 for **a** & **b** and 5 for **c** independent groups of >30 seedlings). Black dots represent individual data points. FW, fresh weight. **d** Electrolyte leakage. Conductivity was measured with rosette leaves from 3-week-old plants immediately after heat treatment at 40 °C for 6 h. Electrolyte leakage was calculated and shown here as % conductivity of total ions (boiled sample). Values are average ± S.D. *P* values indicate significant difference from WT determined by two-tailed student’s *t* test (*n* = 5 leaves from independent plants). Black dots represent individual data points. DKO, double knockout mutant of *GAPC*. **e** Reactive oxygen species production. Rosette leaves from 3-week-old plants were incubated with NBT immediately after heat treatment at 40 °C for 6 h. NBT-formazan was quantified as absorbance at 700 nm and shown here as per tissue weight. Values are average ± S.D. *P* values indicate significant difference from WT determined by two-tailed student’s *t* test (*n* = 5 leaves from independent plants). Black dots represent individual data points. **f** Seedling survival at 45 °C. Five-day-old seedlings were treated at 45 °C for 2.5 h, then placed back to normal temperature condition. After 2 days the seedlings were measured for survival rate. Values are average ± S.D. *P* values indicate significant difference from WT determined by two-tailed student’s *t* test (*n* = 3 independent groups of >30 seedlings). Black dots represent individual data points.

NF-YC10 is involved in heat stress response; its overexpression enhanced the expression of some heat-inducible genes and rendered Arabidopsis seedlings more tolerant to heat stress, while knockdown mutation of *NF-YC10* had the opposite effects^27^. Thus, we tested the effect of altered *GAPC* expression on plant response to heat stress, using a double knockout mutant *gapc1gapc2* and plants overexpressing *GAPC1* or *GAPC2* under the control of the CaMV-35S promoter (*GAPC*1-OE and *GAPC2*-OE) that we previously generated and characterized^2,28^. Those alterations of *GAPC1* or *GAPC2* had no effect on seed germination and seedling growth under normal growth condition (Supplementary Fig. 1A)^7^. When 5-day-old seedlings were treated at 40 °C for varied durations up to 8 h and then transferred back to the normal temperature conditions for recovery for 2 days, all plants showed no observable difference up to 4 h of heat stress (Fig. 3a–c; Supplementary Fig. 1B). However, after 6 h of heat stress, some of the *GAPC*-OE seedlings survived whereas most WT and *gapc1gapc2* seedlings died as indicated by cotyledon bleaching (Supplementary Fig. 1B). Quantitative measurements performed with groups of seedlings indicated that *GAPC1*-OE and *GAPC2*-OE, when compared to WT, began to show increased tolerance to heat stress at 6 h of heat treatment in terms of seedling survival rate, chlorophyll content, and seedling weight (Fig. 3a–c). Although WT and *gapc1gacp2* seedlings were comparable in growth at 40 °C, *gapc1gacp2* seedlings displayed increased electrolyte leakage and reactive oxygen species production (Fig. 3d,e), indicating that plants without GAPCs suffer more severe cell damage than WT under heat stress. The difference in heat tolerance between *GAPC*-altered and WT plants was more obvious when they were subjected to a higher temperature. When 5-day-old seedlings were treated at 45 °C for 2.5 h then returned to normal temperature conditions, more than 60% of *GAPC*-OE seedlings remained alive, whereas only 8% of WT survived and nearly all *gapc1gacp2* seedlings died (Fig. 3f). Taken together, the data suggest that the presence of GAPC is positively associated with heat tolerance in Arabidopsis.

### GAPC regulates the expression of heat-inducible genes

**Fig. 4:**
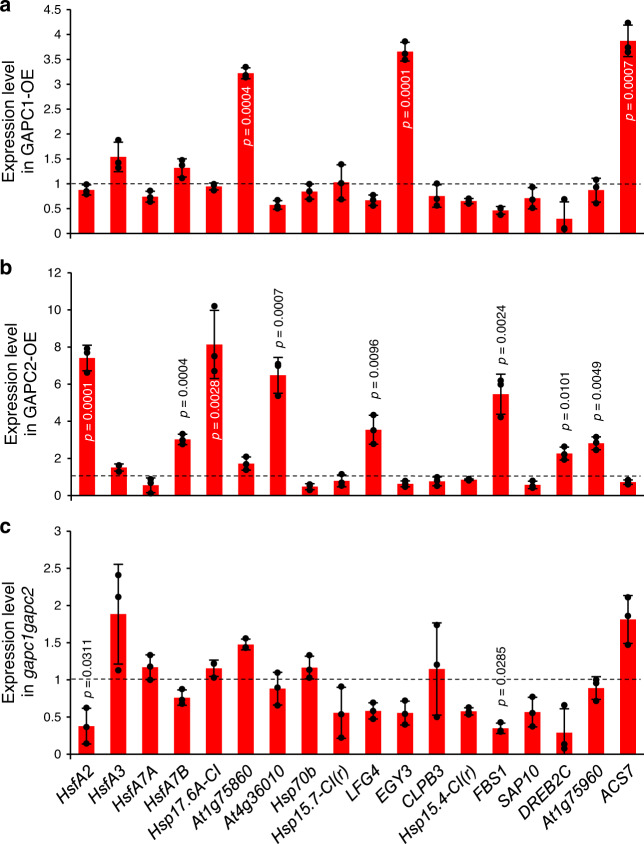
Expression of heat-inducible genes in *GAPC*-altered Arabidopsis. Total RNA was extracted from 5-day-old seedlings of *GAPC1*-OE (**a**), *GAPC2*-OE (**b**), and *gapc1gapc2* (**c**) treated at 37 °C for 5 h and quantitative RT-PCR was performed with gene-specific primers. Values are average ± S.D. and shown as fold change to WT (dashed line). *P* values indicate significant difference from WT determined by two-tailed student’s *t* test (*n* = 3 independent groups of >10 seedlings). Black dots represent individual data points. Hsf, heat shock transcription factor; Hsp, heat shock protein; At1g75860, DNA ligase; At4g36010, pathogenesis-related thaumatin superfamily protein; LFG4, LIFEGUARD4 (Bax inhibitor-1 family protein); EGY3, ETHYLENE-DEPENDENT GRAVITROPISM-DEFICIENT AND YELLOW-GREEN-LIKE3; CLPB3, CASEIN LYTIC PROTEINASE B3; FBS1, F-BOX STRESS INDUCED1; SAP10, STRESS-ASSOCIATED PROTEIN10; DREB2C, DEHYDRATION-RESPONSIVE ELEMENT BINDING PROTEIN 2C; At1g75960, AMP-dependent synthetase and ligase family protein; ACS7, 1-AMINO-CYCLOPROPANE-1-CARBOXYLATE SYNTHASE7.

*NF-YC10* overepxression enhances the expression of some heat-inducible genes^27^. To determine if these NF-YC10 target genes are affected by *GAPC* alterations, we tested *GAPC*-OEs and *gapc1gapc2* for heat-induced expression of 18 heat-inducible genes that were up-regulated by NF-YC10. *GAPC1* and *GAPC2* overexpression increased the transcript levels of different subsets of the heat-inducible genes over those of WT (Fig. 4a, b). *GAPC1*-OE seedlings exhibited an ~3- to 4-fold increase in *At1g75860*, *EGY3* (*ETHYLENE-DEPENDENT GRAVITROPISM-DEFICIENT AND YELLOW-GREEN-LIKE3*), and *ACS7* (*1-AMINO-CYCLOPROPANE-1-CARBOXYLATE SYNTHASE7*) under heat stress, compared to those levels in WT (Fig. 4a). By comparison, *GAPC2*-OE seedlings had increased transcript levels up to 8-fold of 8 heat-inducible genes, *HsfA2* (*heat shock transcription factor A2*), *HsfA7B*, *Hsp17.6A-CI*, *At4g36010* (encoding a pathogenesis-related thaumatin superfamily protein), *LFG4* (*LIFEGUARD4*, a Bax inhibitor1 family protein), *FBS1* (*F-BOX STRESS INDUCED1*), *DREB2C* (*DEHYDRATION-RESPONSIVE ELEMENT BINDING PROTEIN 2C*), and *At1g75960* (encoding an AMP-dependent synthetase and ligase family protein) (Fig. 4b). A slight decrease in the expression of *HsfA2* and *FBS1* was observed in *gapc1gapc2* (Fig. 4c). Expression level of *GAPC* was not significantly affected by overexpression of the other GAPC, and *NF-YC10* expression level remained similar in all *GAPC*-altered plants (Supplementary Fig. 2). The expression of *GAPC1* and *GAPC2* was not induced by heat stress at 38 °C for up to 3 h, as indicated by the data obtained from public microarray database (AtGenExpress)^29^.

### The GAPC-mediated heat responses require NF-YC10

**Fig. 5:**
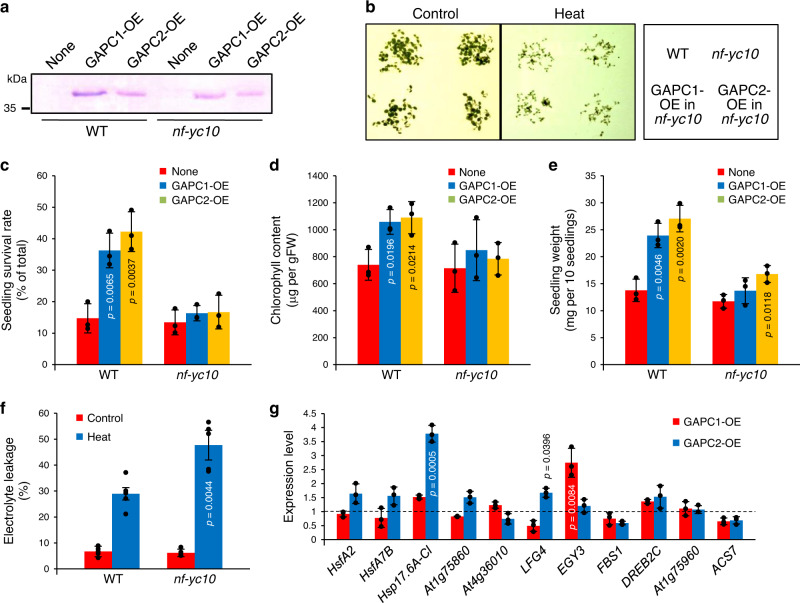
Effects of *GAPC* overexpression in *nf-yc10* on heat response. **a** Immunoblotting of GAPC. Total proteins were extracted from 5-day-old transgenic seedlings overexpressing GAPC-Flag as indicated on the top in the background plants indicated at the bottom. GAPC was probed with an anti-Flag antibody by immunoblotting. **b** Representative images of heat-treated plants. Five-day-old seedlings were untreated (Control) or treated at 40 °C for 6 h, then placed back to normal temperature condition. Images were taken after 2 days of heat shock. **c**–**e** Quantitative measurements of heat response. Plants with genetic background indicated on the x-axis were treated at 40 °C for 6 h and measured for seedling survival rate (**c**), chlorophyll content (**d**), and seedling weight (**e**). Values are average ± S.D. *P* values indicate significant difference from background line (None) determined by two-tailed student’s *t* test (*n* = 3 independent groups of >30 seedlings). Black dots represent individual data points. FW, fresh weight. **f** Electrolyte leakage of WT and *nf-yc10*. Conductivity was measured with rosette leaves from 3-week-old plants immediately after heat treatment at 40 °C for 6 h. Electrolyte leakage was calculated and shown here as % conductivity of total ions (boiled sample). Values are average ± S.D. *P* values indicate significant difference from WT determined by two-tailed student’s *t* test (*n* = 5 leaves from independent plants). Black dots represent individual data points. **g** Expression of heat-inducible genes. Total RNA was extracted from 5-day-old seedlings treated at 37 °C for 5 h and quantitative RT-PCR was performed with gene-specific primers. Values are average ± S.D. and shown as fold change to background line (*nf-yc10*; dashed line). *P* values indicate significant difference from *nf-yc10* determined by two-tailed student’s *t* test (*n* = 3 independent groups of >10 seedlings). Black dots represent individual data points.

To determine if the up-regulation of heat-inducible genes and the enhanced heat tolerance observed in *GAPC*-OE are dependent on the presence of NF-YC10, we overexpressed *GAPC* in *NF-YC10* knockout mutant (*nf-yc10*) and tested the transgenic plants for the expression levels of the 11 heat-inducible genes up-regulated by *GAPC* overexpression in WT and growth phenotype in response to heat. Protein abundance increased by *GAPC* overexpression in both WT and *nf-yc10* backgrounds was confirmed by immunoblotting (Fig. 5a). The *GAPC-*OE in *nf-yc10* (*GAPC-*OE_*nf-yc10*_) plants exhibited growth phenotype indistinguishable from their background plant *nf-yc10* under heat stress (Fig. 5b). Seedling survival rate, chlorophyll content, and seedling weight were not significantly increased by *GAPC* overexpression in *nf-yc10* at 40 °C for 6 h, while still increased by *GAPC* overexpressed in WT (*GAPC*-OE_WT_; Fig. 5c–e). When compared to WT, *nf-yc10* showed an increase in heat-induced electrolyte leakage as did *gapc1gapc2* seedlings at 40 °C (Fig. 5f). The *GAPC-*OE_*nf-yc10*_ plants, when compared with the *nf-yc10* plants, showed no or substantially attenuated heat induction of the genes that were highly up-regulated in *GAPC*-OE_WT_ (Fig. 5g). *GAPC1-*OE_*nf-yc10*_ seedlings showed no increased transcript levels of *At1g75860* and *ACS7* that were up-regulated in *GAPC1-*OE_WT_ under heat stress (Fig. 4a vs. Fig. 5g). The transcript level of *EGY3* was increased in *GAPC1-*OE_*nf-yc10*_ but the magnitude of increase was smaller than that in *GAPC1-*OE_WT_, compared to their respective background *nf-yc10* and WT seedlings (Fig. 4a vs. Fig. 5g)*. GAPC2-*OE_*nf-yc10*_ seedlings had no increased transcript level of 6 genes up-regulated in *GAPC2-*OE_WT_ under heat stress (Fig. 4b vs. Fig.  5g). The magnitude of increase in *Hsp17.6A-CI* and *LFG4* transcripts was ~2-fold less than that in *GAPC2-*OE_WT_ (Fig. 4b vs. Fig. 5g). Taken together, the results suggest that the GAPC effect on heat tolerance requires the presence of NF-YC10, and that NF-YC10 may function as a downstream target of GAPC in the signaling pathway for plant response to heat stress.

### Heat stress promotes GAPC accumulation in nuclei

**Fig. 6:**
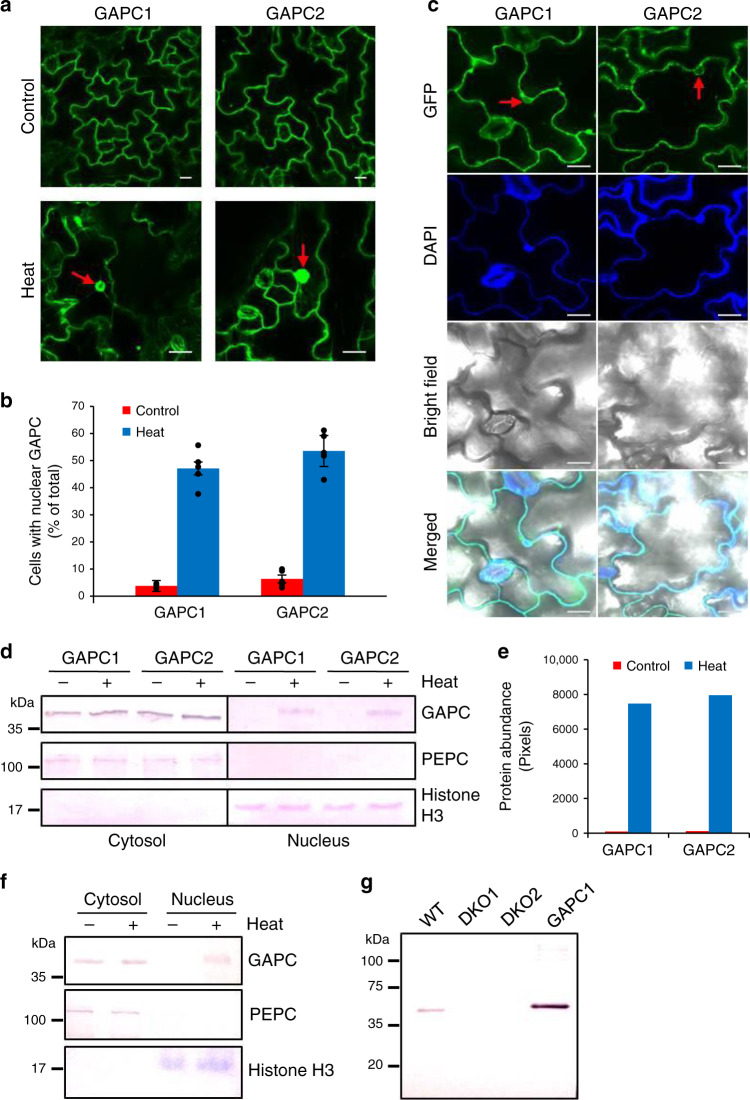
Heat-induced nuclear accumulation of GAPC. **a** Fluorescence image of Arabidopsis leaf cells. Five-day-old transgenic seedlings overexpressing GAPC-GFP were untreated (Control) or treated at 40 °C for 6 h and observed under a confocal microscope. Arrows indicate the nucleus. Scale bars = 10 µm. **b** The number of cells with nuclear GAPC. Plants were treated and observed as in **a**. Cells with clear fluorescence in the nucleus were counted and shown here as % of total cells counted. Values are average ± S.D. from 5 leaves independently treated. Black dots represent individual data points. **c** Confirmation of the nucleus. The heat-treated seedlings were stained with 4′,6-diamidino-2-phenylindole (DAPI) for nuclear counterstaining and observed under a confocal microscope. Shown are representative images taken at the same focal plane. Arrows indicate the nucleus. Scale bars = 10 µm. **d** Immununoblotting of GAPC in cytosolic and nuclear fractions. Five-day-old transgenic seedlings overexpressing GAPC-Flag were untreated (−) or treated at 40 °C for 6 h (+), and cytosolic and nuclear fractions were separated. GAPC was probed with an anti-Flag antibody by immunoblotting. Phosphoenolpyruvate carboxylase (PEPC) and histone H3 were used as cytosolic and nuclear markers, respectively. **e** Quantification of nuclear GAPC. Protein band intensity of GAPC in the nuclear fraction in **d** was measured by ImageJ software. **f** Immununoblotting of endogenous GAPC in cytosolic and nuclear fractions. Five-day-old wild type seedlings were untreated (−) or treated at 40 °C for 6 h (+), and cytosolic and nuclear fractions were separated. GAPC was probed with an anti-GAPC antibody by immunoblotting. PEPC and histone H3 were used as cytosolic and nuclear markers, respectively. **g** GAPC antibody test for specificity. Total proteins were extracted from 5-day-old seedlings indicated on the top. GAPC was probed with the anti-GAPC antibody used in **f** by immunoblotting. Protein marker size is on the left. WT, wild type; DKO, double knockout mutant of *GAPC*; GAPC1, recombinant GAPC1 purified from *E. coli* used as a control.

We then tested whether, in response to heat, the cytosolic GAPCs might enter the nucleus where they could interact with the transcription factor NF-YC10. Arabidopsis transgenic lines overexpressing *GAPC1* or *GAPC2* fused with the green fluorescence protein (GAPC-GFP) were generated to observe their subcellular location. Under normal laboratory temperatures (23 °C), the fluorescence signal was observed mostly in the cytosol for both GAPC1 and GAPC2 (Fig. 6a, Control). After heat treatment at 40 °C for 6 h, however, significant GFP signal was detected in the nucleus of many leaf epidermal cells for both GAPC1 and GAPC2 (Fig. 6a, Heat and 6b). The association of GAPC-GFP signal with the nucleus was confirmed by counterstaining with the nucleic acid-specific 4′,6-diamidino-2-phenylindole (DAPI; Fig. 6c). Nuclear accumulation of other cytosolic glycolytic enzymes, such as non-phosphorylating GAPDH (NP-GAPDH) and hexokinase 1 (HXK1), was not observed in heat-treated transgenic seedlings overexpressing those as a GFP-fusion protein (Supplementary Fig. 3). The heat-induced accumulation of GAPC in the nucleus was also observed with nuclear proteins extracted from heat-treated *GAPC*-OE plants followed by immunoblotting analysis. GAPC1 and GAPC2 proteins were detected in the cytosol regardless of heat treatment, whereas they were found in the nucleus only upon heat stress (Fig. 6d, e). Successful separation of the two fractions was confirmed by probing organelle-specific marker proteins (PEPC for cytosol and histone H3 for nucleus; Fig. 6d).

To test whether endogenous GAPC displays heat-induced nuclear localization, we isolated nuclei from heat-treated WT Arabidopsis and performed immunoblotting using a GAPC-specific antibody. GAPC was present in the nuclei isolated from heat-treated plants, but not in the nuclei from untreated control (Fig. 6f). The antibody was specific to GAPCs because it detected the GAPC band from WT but not from *gapc1gapc2* protein extracts (Fig. 6g). Those results indicate that heat stress promotes the nuclear translocation of endogenous GAPC protein molecules from the cytosol.

### The GAPC-mediated responses require its nuclear accumulation

**Fig. 7:**
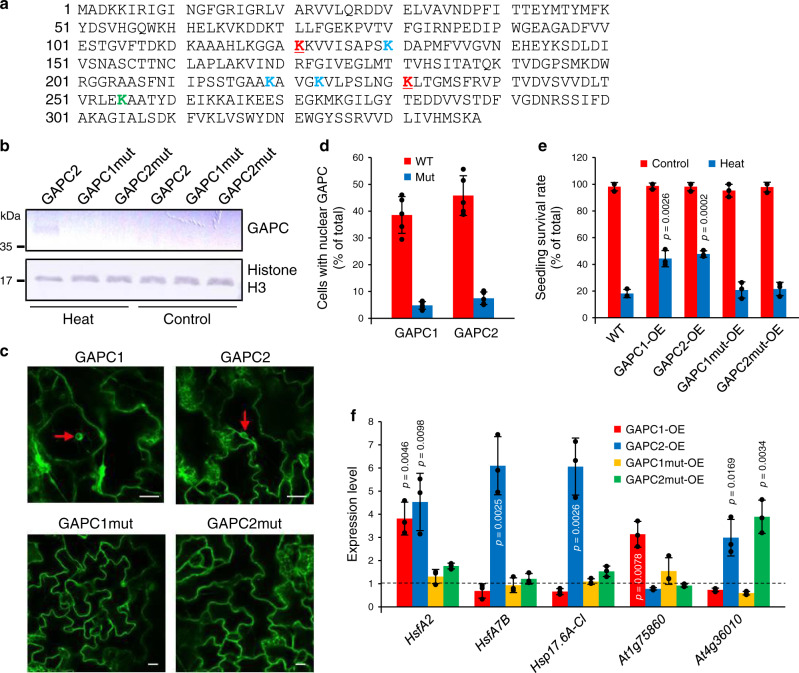
Effects of GAPC mutation on heat response. **a** GAPC2 amino acid sequence. Colored are the lysine residues required for HsGAPDH nuclear localization (K121 and K231 in red, K255 in green) and found to be acetylated in *E. coli* (K130, K219, and K223 in blue, K255 in green). The lysines mutated in this study are underlined. **b** Immununoblotting of GAPC in nuclear fraction. Five-day-old transgenic seedlings overexpressing the indicated GAPC-Flag were untreated (Control) or treated at 40 °C for 6 h. Nuclei were isolated and GAPC was probed with an anti-Flag antibody by immunoblotting. Histone H3 was used as a nuclear marker. GAPCmut, GAPC with K121A/K231A. **c** Fluorescence images of Arabidopsis leaf cells. Five-day-old seedlings overexpressing the indicated GAPC-GFP were treated at 40 °C for 6 h and observed under a confocal microscope. Arrows indicate the nucleus. Scale bars = 10 µm. **d** The number of cells with nuclear GAPC. Plants were treated and observed as in **c**. Cells with clear fluorescence in the nucleus were counted and shown here as % of total cells counted. Values are average ± S.D. from 5 leaves independently treated. Black dots represent individual data points. **e** Quantitative measurement of heat response. Plants were untreated (Control) or treated at 40 °C for 6 h and measured for seedling survival rate. Values are average ± S.D. *P* values indicate significant difference from WT determined by two-tailed student’s *t* test (*n* = 3 independent groups of >30 seedlings). Black dots represent individual data points. **f** Expression of heat-inducible genes. Total RNA was extracted from 5-day-old seedlings treated at 37 °C for 5 h and quantitative RT-PCR was performed with gene-specific primers. Values are average ± S.D. and shown as fold change to WT (dashed line). *P* values indicate significant difference from WT determined by two-tailed student’s *t* test (*n* = 3 independent groups of >10 seedlings). Black dots represent individual data points.

We next decided to see if interference with the GAPC nuclear localization compromises its ability to control the heat tolerance of seedlings and the expression of heat-inducible genes. While GAPDH, including GAPCs, has no nuclear localization sequence, human GAPDH (HsGAPDH) has three lysine residues (K117, K227, and K251) whose acetylation is necessary and sufficient for apoptotic stress-induced nuclear translocation of HsGAPDH^30^. The three lysines are conserved in Arabidopsis GAPC at K121, K231, and K255 (Fig. 7a). In addition, GAPC2 was reported to be acetylated at four lysine residues (K130, K219, K223, and K255) when expressed and purified in *E. coli*^31^. Thus, we substituted several combinations of the six lysines (K255 overlapping) to alanines in both GAPC1 and GAPC2 and overexpressed the various lysine-mutated proteins in Arabidopsis (Fig. 7a). We found that only one combination of the lysine mutations (K121A and K231A; GAPCmut) compromised the heat-induced nuclear accumulation of both GAPC1 and GAPC2, as revealed by immunoblotting of nuclei isolated from the transgenic plants (Fig. 7b; Supplementary Fig. 4A) and confocal microscopic analysis of GFP-fused GAPCmut (Fig. 7c, d). Analyses of seedling survival under heat stress condition and the gene expression profiling demonstrated that when overexpressed and compared to WT, both GAPC1mut and GAPC2mut failed to enhance heat tolerance of growth (Fig. 7e) and increase the expression of some heat-inducible genes that were observed to be up-regulated by the intact *GAPC* overexpression (Fig. 7f). There was no significant difference in catalytic activity between GAPCmut and intact GAPC (Supplementary Fig. 4B). These data suggest that GAPC is required to translocate into the nucleus to affect NF-YC10-mediated heat responses observed in *GAPC*-OE_WT_.

### The heat responses require the GAPC and NF-YC10 interaction

**Fig. 8:**
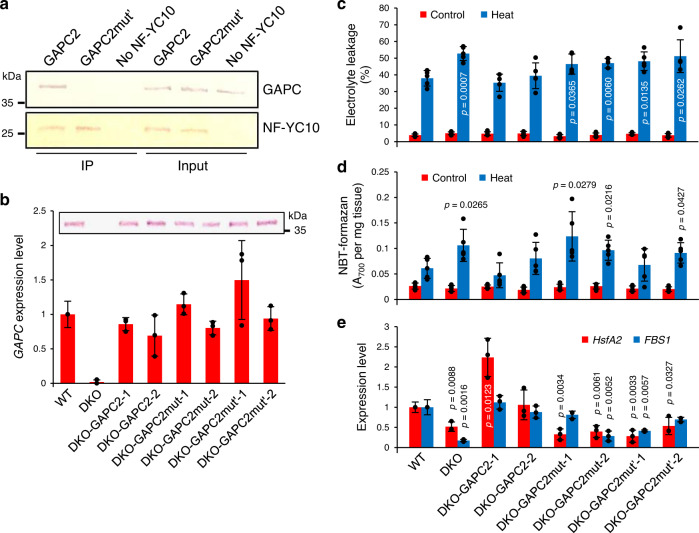
Heat response of *gapc1gapc2* (DKO) complemented with *GAPC2* variants. **a** Co-immunoprecipitation of GAPC and NF-YC10. Purified GAPC2-6xHis or GAPC2mut’-6xHis was mixed with NF-YC10-6xHis-STREP. Immunoprecipitation (IP) was performed using an anti-STREP antibody, then immunoblotting was performed using an anti-6xHis antibody. Protein input is shown on the right. **b** *GAPC* expression levels. Total RNA was extracted from 10-day-old seedlings and quantitative RT-PCR was performed with *GAPC2*-specific primers. Values are average ± S.D. from 3 independent seedlings and shown as fold change to WT. Black dots represent individual data points. DKO, double knockout mutant of *GAPC*. Inset, immunoblotting of GAPC. Total proteins were extracted from 5-day-old seedlings and GAPC was probed with the anti-GAPC antibody. **c** Electrolyte leakage. Conductivity was measured with true leaves from 2-week-old plants immediately after heat treatment at 40 °C for 6 h. Electrolyte leakage was calculated and shown here as % conductivity of total ions (boiled sample). Values are average ± S.D. *P* values indicate significantly higher than WT determined by two-tailed student’s *t* test (*n* = 5 leaves from independent plants). Black dots represent individual data points. **d** Reactive oxygen species production. Rosette leaves from 3-week-old plants were incubated with NBT immediately after heat treatment at 40 °C for 6 h. NBT-formazan was quantified as absorbance at 700 nm and shown here as per tissue weight. Values are average ± S.D. *P* values indicate significant difference from WT determined by two-tailed student’s *t* test (*n* = 5 leaves from independent plants). Black dots represent individual data points. **e** Expression of heat-inducible genes. Total RNA was extracted from 10-day-old seedlings treated at 37 °C for 5 h and quantitative RT-PCR was performed with gene-specific primers. Values are average ± S.D. and shown as fold change to WT. *P* values indicate significantly lower than WT determined by two-tailed student’s *t* test (*n* = 3 independent seedlings). Black dots represent individual data points.

The above results of GAPC-enhanced heat responses were obtained mostly from ectopic overexpression of *GAPC* by the constitutive CaMV-35S promoter. To determine the physiological relevance of these observations in native plants, we generated two independent lines of *gapc1gapc2* plants genetically complemented with *GAPC2* or *GAPC2mut* under its native promoter, and compared their heat phenotype and the expression of heat-inducible genes with those of *gapc1gapc2*. In addition, to test whether the GAPC effect is dependent on the GAPC and NF-YC10 interaction in nuclei, we identified a GAPC2mut’ that does not bind NF-YC10 but still underwent nuclear translocation under heat stress. The lysine-mutated GAPCmut’ protein (K121/219/223/255A) failed to bind NF-YC10, as indicated by the lack of co-immunoprecipitation (Fig. 8a). Hence, *GAPC2mut’*-complemented *gapc1gapc2* was included to examine the effect of GAPC-NF-YC10 interaction, as well as GAPC nuclear accumulation, on the heat responses of *gapc1gapc2*. A significant increase in the expression of *GAPC2* and its protein product in all the complemented plants was confirmed by qRT-PCR and immunoblotting, respectively (Fig. 8b). While the heat-induced electrolyte leakage and reactive oxygen species production in *GAPC2*-complemented *gapc1gapc2* were fully restored to the level of WT, that of *GAPC2mut*- or *GAPC2mut’*-complemented *gapc1gapc2* still remained significantly higher than WT (Fig. 8c, d). Likewise, the expression of the two heat-inducible genes observed to be down-regulated in *gapc1gapc2* (*HsfA2* and *FBS1*; Fig. 4c) was recovered in *GAPC2*-complemented *gapc1gapc2*, but not in *GAPC2mut*- or *GAPC2mut’*-complemented *gapc1gapc2* (Fig. 8e). Restoration of the cytosolic function of GAPC in the complemented plants was confirmed by measuring the catalytic activity of GAPC (Supplementary Fig. 5). Together with the heat-induced nuclear accumulation of endogenous GAPC (Fig. 6f), these data indicate that both GAPC nuclear localization and NF-YC10 interaction are required for heat tolerance in native plants, not only upon *GAPC* overexpression. The full recovery by *GAPC2* complementation also verifies that the heat sensitivity observed in *gapc1gapc2* (Figs. 3 and 4) is due to *GAPC* disruption.

### GAPC enhances DNA binding ability of NF-YC10

**Fig. 9:**
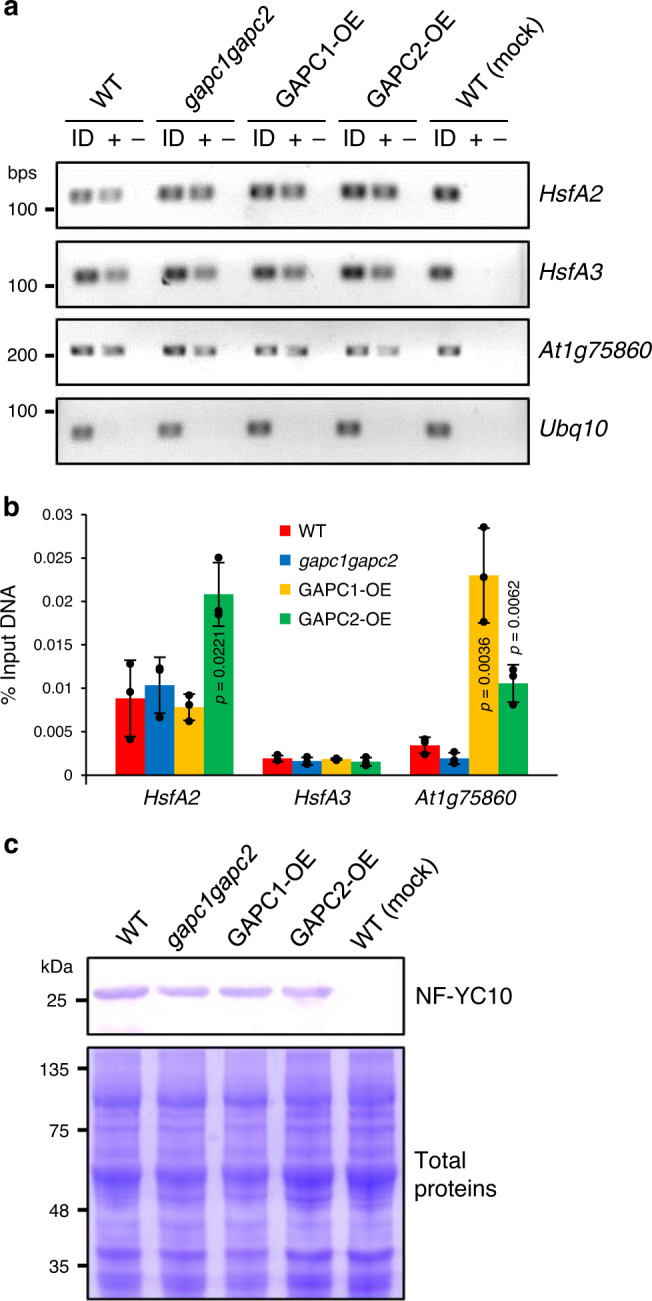
Effect of GAPC on NF-YC10 binding to its target promoters. **a** PCR verification of the precipitated DNA. Chromatin immunoprecipitation (ChIP) was performed using anti-Flag antibody from heat-treated mesophyll protoplasts that were isolated from the *GAPC*-altered Arabidopsis lines indicated on the top and transfected with *35S:NF-YC10-Flag*. Input DNA (ID; *see* Methods for details) and DNA precipitated with (+) or without (−) the antibody were used for PCR with primers specific to the promoters of genes indicated on the right. Mock, non-transfected; *Ubq10*, ubiquitin10. **b** Quantification of the precipitated DNA. Quantitative PCR was performed with the DNA samples obtained from **a**. Values are average ± S.D. and shown as % of PCR product amplified from the input DNA. *P* values indicate significant difference from WT determined by two-tailed student’s *t* test (*n* = 3 independent pools of protoplasts). Black dots represent individual data points. **c** NF-YC10 protein abundance. After transfection, total proteins were extracted from the protoplasts and immunoblotting was performed with anti-Flag antibody (top). Coomassie blue staining is shown for loading control (bottom).

The expression of some NF-YC10 target genes increased by *GAPC* overexpression led us to see whether GAPC could affect the ability of NF-YC10 to bind its target promoters. Sato et al. confirmed experimentally that NF-YC10 bound directly to the promoters of *HsfA2*, *HsfA3*, and *At1g75860*^27^. Thus, to verify GAPC effects on DNA binding ability of NF-YC10 to these promoters, we performed chromatin immunoprecipitation (ChIP) using mesophyll protoplasts that were isolated from the *GAPC*-altered Arabidopsis lines. The protoplasts were transfected with *35S:NF-YC10-Flag*, then heat-treated at 37 °C for 5 h. When ChIP was carried out with an anti-Flag antibody, the successful isolation of all three gene’s promoter regions associated with NF-YC10 was confirmed by subsequent PCR amplification (Fig. 9a). No PCR product was detected when the antibody was omitted or when WT protoplasts were not transfected (mock; Fig. 9a). A negative control gene (*ubiquitin10*) showing no DNA band confirmed the specificity of NF-YC10 binding to the heat-inducible genes (Fig. 9a). Quantitative PCR analysis revealed that when compared to those found in WT, *HsfA2* and *At1g75860* promoter regions were highly enriched in *GAPC2*-OE and *GAPC1*-OE protoplasts respectively, with no significant change in *HsfA3* promoter region precipitated with NF-YC10 (Fig. 9b). These results are in agreement with the increased expression of *HsfA2* and *At1g75860* in *GAPC2*-OE and *GAPC1*-OE seedlings, respectively (Fig. 4a, b). We verified by immunoblotting using the anti-Flag antibody that NF-YC10 was expressed comparably among the different samples (Fig. 9c), indicating that the DNA enrichment observed in *GAPC*-OE protoplasts was independent of NF-YC10 protein abundance.

## Discussion

**Fig. 10:**
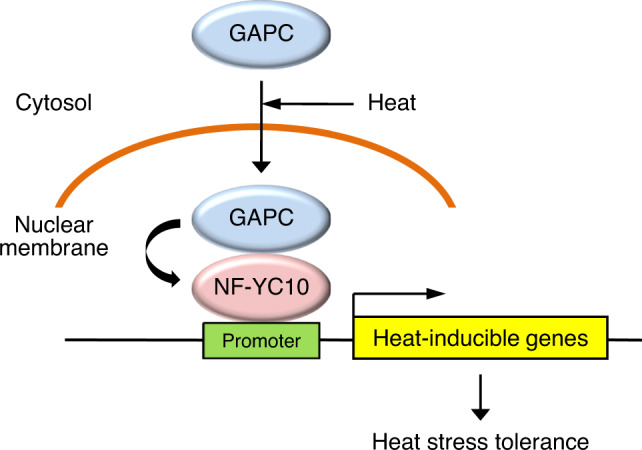
Proposed model for GAPC-mediated heat response in Arabidopsis. GAPC that normally exists in the cytosol enters the nucleus in response to heat. In the nucleus GAPC binds to the transcription factor NF-YC10 to increase the expression of heat-inducible genes, rendering Arabidopsis tolerant to heat stress.

Enzyme moonlighting is a phenomenon whereby an enzyme can perform secondary non-enzymatic functions acquired through evolution, such as transcriptional regulation, signal transduction, and apoptosis, in addition to its canonical catalytic function^19^. The glycolytic enzyme GAPC is a moonlighting enzyme and plays important roles in plant response to stress, but its role in the nucleus in mediating stress response was unclear. Based on our data presented in this study, we propose that heat stress promotes translocation of a portion of the cytoplasmic GAPC pool into Arabidopsis nuclei, where GAPC is associated with the transcription factor NF-YC10 to increase the expression of target heat-inducible genes and plant heat tolerance (Fig. 10). These findings show that GAPC regulates transcriptional activity by directly interacting with a specific transcription factor in plants.

Nuclear factor Y (NF-Y) is an evolutionarily conserved transcription factor family composed of three subunits, NF-YA, NF-YB, and NF-YC, which can form functional heterodimers or heterotrimers with one another in a heat-dependent manner^32^. Plant genomes have multiple members encoding each NF-Y subunit (10 genes for each subunit in Arabidopsis), enabling a wide variety of heteromeric combinations and potentially diverse functions. In addition, NF-Y subunits can also interact with other proteins in various kinds of complexes. Particularly, NF-YC10, also known as DNA POLYMERASE II SUBUNIT B3-1 (DPB3-1), binds to DEHYDRATION RESPONSE ELEMENT-BINDING PROTEIN 2A (DREB2A) and forms a heterotrimer with NF-YA2 and NF-YB3 in a heat stress-dependent manner in Arabidopsis^27^. The heterotrimer forms a transcriptional complex with DREB2A at the promoters of heat-inducible DREB2A target genes, thus enhancing the efficiency of transcription by DREB2A^27^. In this regard, GAPC interaction with NF-YC10 might affect the structural integrity and/or regulatory activity of the transcriptional complex to regulate the expression of heat-inducible genes. However, our co-immunoprecipitation assays demonstrated that GAPC neither directly bound to the components of the transcriptional complex, including DREB2A, nor noticeably affected NF-YC10 interaction with the individual binding partners (Supplementary Fig. 6). While these analyses are limited to in vitro conditions, other approaches considering post-translational modification and nuclear elements that might come into play could uncover the effect of GAPC on the NF-Y heterocomplex. Meanwhile, *GAPC* overexpression increased the expression of other heat-inducible genes that were not DREB2A target genes, as well as the genes downstream of DREB2A. Indeed, the expression of *HsfA2*, which is not a DREB2A target gene, is increased by *NF-YC10* overexpression and its promoter is directly bound by NF-YC10 under heat stress^27^. This implies that NF-YC10 can enhance the expression of heat-inducible genes through DREB2A-independent pathways as well. These data, together with our results from ChIP assay, suggest that GAPC affects the ability of NF-YC10 itself to bind its target promoters, such as that of *HsfA2*. Although GAPC-DNA interaction has been reported^13,16^, direct interaction of GAPC with any regulatory elements of the heat-inducible genes can be ruled out by our observation of no or marginal heat induction of the genes in *GAPC*-OE_*nf-yc10*_. Therefore, it should be through the direct interaction with NF-YC10 that GAPC increases the expression of the heat-inducible genes. It should be noted that the transcription factor library screened for GAPC interaction did not contain all transcription factors in Arabidopsis^24^, and thus GAPC may interact with other transcription factors under stress.

Our data show that the presence of GAPC in the nucleus is induced by heat stress. As described earlier, GAPC can undergo different conditional post-translational modifications on its highly reactive cysteine residue, which possibly determines some of its moonlighting functions. Post-translational modification is speculated as a key initial process leading to stress-dependent nuclear translocation of GAPC. *S*-nitrosylation might be important because *S*-nitrosylation level of GAPC was transiently increased in salt-treated tobacco BY-2 cells, and mutation of the active cysteines into serines impaired salt-dependent GAPC interaction with osmotic stress-activated protein kinase (OSAK) in the nucleus but not in the cytoplasm^33^. However, both cadmium- and long-chain base-induced nuclear translocation of GAPC observed in Arabidopsis and tobacco, respectively, were independent of *S*-nitrosylation^6,15^. A reduced nuclear localization of GAPC was observed in Arabidopsis *des1* mutant defective in L-cysteine desulfhydrase DES1 and this was recovered by exogenous sulfide treatment^14^. Together with detection of a sulfhydryl modification of catalytic cysteine in nuclear GAPC1^14^, this suggests that *S*-sulfhydration is a possible mechanism for GAPC nuclear localization. It is reported that oxidative stress by H_2_O_2_ treatment also induces nuclear localization of GAPC by modifying the catalytic cysteine (C156)^5^, discrepant with another report that C156S had no effect on GAPC nuclear translocation by cadmium-induced oxidative stress^6^. Thus, different stress signals may differentially modify GAPC on its active site cysteine residues, as observed for those of animal GAPDH.

Interestingly, under apoptotic stressors, acetylation at three lysines (K117, K227, and K251 in HsGAPDH) is required for human GAPDH translocation into the nucleus^30^. The importance of lysine acetylation for stress-induced GAPC nuclear localization was also shown in rice subjected to oxidative stress^16^. However, none of the lysines identified to be responsible for rice GAPC nuclear localization (K57, K74, and K217 in OsGAPDH1) corresponded to those for HsGAPDH^16^. Although the three lysines in HsGAPDH and OsGAPDH1 are absolutely conserved in Arabidopsis GAPC, only one of them (K76 in AtGAPC) is acetylated in unstressed Arabidopsis from which K76, K130, K198, K263, K267, and K310 found to be acetylated^34^. Moreover, our data presented here indicate that two lysines (K121 and K231 in AtGAPC), which are necessary for HsGAPDH nuclear translocation but neither acetylated in Arabidopsis nor required for OsGAPDH1 nuclear localization, are sufficient for the GAPC nuclear localization in response to heat. These signify the lysine acetylation requirement for stress-induced nuclear translocation conserved between animal and plant GAPDHs, but differential post-translational modifications for the GAPC subcellular distribution even within the plant kingdom. Recently, an E3 ubiquitin-protein ligase SEVEN IN ABSENTIA like 7 (SINAL7) was found to directly interact with GAPC1 in vitro, and nuclear GAPC abundance was decreased in *sinal7* and increased by *SINAL7* overexpression^17,35^. Interestingly, K231A mutation in GAPC1 abolished its interaction with SINAL7, and K76 in GAPC1 corresponding to K74 in OsGAPDH1 was ubiquitinated by SINAL7^17^. *SINAL7* overexpression enhanced drought tolerance of Arabidopsis, increased *DREB2A* expression, and decreased stomatal aperture^35^. These data suggest a potential role of ubiquitination, particularly through K231, in heat-dependent nuclear translocation of GAPC. Taken together, different extracellular stimuli and their intracellular signaling messengers (e.g. heat, salt, osmolyte, heavy metal, reactive oxygen species, and lipid mediators) might differentially and specifically modify GAPC for nuclear access, possibly through cysteine sulfur-modification, lysine acetylation, ubiquitination, or yet unidentified post-translational modifications.

Another intriguing question is the metabolic implication of the nuclear translocation of GAPC under heat stress. Unlike animal GAPDH with only one cytosolic form, GAPDHs in higher plants are classified into three groups according to their subcellular locations: cytoplasmic GAPC for glycolysis, glycolytic GAPCp in non-green plastids, and chloroplastic GAPA/B for photosynthetic CO_2_ fixation^1,36–38^. In addition, the Arabidopsis cytoplasm contains non-phosphorylating (NP)-GAPDH that catalyzes oxidation, instead of phosphorylation, of the substrate for cytoplasmic maintenance of NADPH^39^. Our previous analysis showed that the double knock of *GAPC1* and *GAPC2* resulted in significant changes in the level of glycolytic intermediates and the ratios of ATP/ADP and NAD(P)H/NAD(P)^28^. However, the *GAPC*-deficient plants display no overt changes in vegetative growth, but decreased seed oil content compared to WT^2,28^. This complexity of the plant GAPDH families and tolerance of Arabidopsis plants to metabolic perturbation caused by *GAPC* alterations could be explained in part by the partial genetic redundancy of plant *GAPDH*. Under heat stress, only a small fraction of GAPC was translocated into nuclei based on our immunoblotting and microscopic analyses. Thus, the heat-induced nuclear translocation of GAPC might not drastically decrease the cytosolic glycolytic activity, and instead the major function of the stress-induced nuclear GAPCs is via regulation, including gene expression as shown in this study. However, heat stress may reduce the overall cytosolic glycolytic activity because GAPCs are potentially inactivated by the stress-induced oxidation. GAPDH, including GAPC, has been proposed to function as a redox sensor through its highly reactive cysteine for the regulation of energy metabolism^5^. In animal system, oxidative inactivation of GAPDH enables cells to redirect the carbohydrate metabolic flux from glycolysis to pentose phosphate pathway for NAPDH production^40,41^.

Moonlighting of metabolic enzymes, such as GAPDH, provides a mechanistic link between metabolic activity and cellular regulation, and dysfunction of such regulation has severe physiological and pathological consequences in animal systems. Although the stress-induced nuclear moonlighting of GAPC has been described in many studies, downstream signaling target(s) and GAPC effects on its/their nuclear function have largely been unexplored. By demonstrating a heat-dependent action of GAPC in the nucleus, we show the apparent bifurcating property of plant GAPC that depends distinctively on catalytic activity for constitutive cellular process and on subcellular localization for stress response. Hence, our study brings significance in understanding the energy-efficient strategy that cells have developed to increase functional options without increasing the number of genes to avoid the highly energy-consuming processes, replication, transcription, and translation. Furthermore, our study could represent future directions to improve heat tolerance of crops by providing a molecular basis to adjust the balance between normal plant growth and stress response, as heat stress is an increasing problem for crop cultivation and food security.

## Methods

### Identification of GAPC-binding transcription factors

The transcription factor cDNA library was previously cloned and expressed in *E. coli*^42^. Briefly, a library containing 1498 individually cloned cDNA of Arabidopsis transcription factors^24^ was cloned into pDONR221^TM^ then pET-53-DEST^TM^ with 6xHis tag (Novagen) using Gateway® Recombination Kit (Life Technologies), and was transformed into Max Efficiency® DH5α Competent Cells (Life Technologies) for plasmid propagation then into *E. coli* Rosetta^TM^ (DE3) for protein expression. Sequence of primers used for cloning is provided in Supplementary Table 1. Based on estimate by Clarke and Carbon (1976)^43^, we calculated the number of colonies required to cover 1498 different clones as *N* = ln(1-*P*)/ln(1-*F*), where *P* = desired probability of finding the clone of interest (typically 0.99) and *F* = fractional portion of the library present in a single clone (1/1498). *N* was estimated at a maximum of 6900, so we used over 7000 colonies for protein extraction. Colonies were pooled and incubated in Luria-Bertani (LB) media at 15 °C for 6 h for induction. Proteins were purified using Ni-nitrilotriacetic acid (NTA) Agarose (QIAGEN). Fifty μg of the purified proteins were co-immunoprecipitated as described below with GAPC2-Flag isolated from Arabidopsis overexpressing the recombinant protein or from control plants with empty vector (EV) using an anti-Flag antibody (GenScript A00187; 1:100 diluted). The GAPC2-bound proteins were resolved by SDS-PAGE and visualized by Coomassie blue. Each whole lane containing protein bands was carefully excised from the gel and divided into several pieces, and the proteins were in-gel digested with trypsin (Sigma-Aldrich) at 37 °C overnight, according to the manufacturer’s instructions. The digested peptides were run on the LC-tandem MS using an Orbitrap Fusion Lumos (Thermo Scientific) coupled with a Dionex RSLCnano HPLC (Thermo Scientific). The samples were dried down to concentrate and resuspended in 10 μL of 5% (v/v) acetonitrile/0.1% (v/v) formic acid mixture. 5 μL was injected for LC-MS/MS on 2-h gradient separation and data acquisition. Peptides were resolved using 75 μm × 50 cm PepMap C18 column (Thermo Scientific). The database search was performed with peptide mass fingerprint data using Mascot (v2.5.1.0) database search engine (Matrix Science) against the *Arabidopsis thaliana* proteome database (Uniprot.org). Mascot was searched with a fragment ion mass tolerance of 0.60 Da and a parent ion tolerance of 10 ppm. The criteria for a significant protein identification were both at least two unique peptides per protein identified and each peptide showing a probability >99%. Data are available via ProteomeXchange with identifier PXD018945 [10.6019/PXD018945]. Proteins found from GAPC2 but not from EV (designated as ‘S’ and ‘C’ in the data repository, respectively) were considered as GAPC-binding candidates.

### Plant materials and growth condition

All Arabidopsis plants used were Columbia-0 ecotype of *Arabidopsis thaliana*. T-DNA insertional knockout mutants were obtained from the Arabidopsis Biological Resource Center (Ohio State University) and homozygotes were confirmed by PCR-based genotyping. All *GAPC* transgenic plants and *GAPC* double knockout mutants (*gapc1gapc2*) were previously generated, and the floral dipping method was applied to generate all other transgenic plants used in this study^2,28^. Seeds were surface-sterilized with 70% (v/v) ethanol, and then 20% (v/v) bleach, followed by washing 5 times with water. Sterilized seeds were stratified at 4 °C for 2 days prior to germination. Plants were germinated and grown on 1/2 strength of Murashige and Skoog (MS) plates with 1% (w/v) sucrose and 0.8% (w/v) agar at 22 °C under light cycles of 16-h light/8-h dark.

### Immunoprecipitation

Immunoprecipitation was performed using an anti-Flag antibody (GenScript A00187; 1:100 diluted) or anti-STREP antibody (GenScript A00626; 1:100 diluted) and Protein A-agarose (Sigma-Aldrich), according to the manufacturer’s instructions. GAPCs were Flag-tagged whereas NF-YC10 was STREP-tagged. Plant tissues were ground with liquid nitrogen and incubated in protein extraction buffer (50 mM Tris-HCl pH7.3, 50 mM NaCl, 5% (v/v) glycerol, 1 mM DTT) containing a protease inhibitor cocktail (Sigma-Aldrich) on ice for 30 min. Supernatant after centrifugation at 12000 × g for 10 min at 4 °C was used as a protein extract. 10 mg of Protein A-agarose was swollen with 0.2 mL of buffer A (20 mM NaH_2_PO_4_ pH8.0, 150 mM NaCl) for 1 h and washed with buffer A. The protein extract was mixed with 5 μg of antibody, the swollen Protein A-agarose, and buffer A to the final volume of 1 mL, and gently agitated overnight at 4 °C. The mixture was washed five times with buffer A by centrifugation at 2500 × *g* for 1 min. Target proteins were added to the immunocomplex and gently agitated overnight at 4 °C. The mixture was washed five times with buffer A by centrifugation at 2500 × *g* for 1 min. SDS-PAGE loading buffer was added and boiled for 10 min. After brief centrifugation, the supernatant was used for SDS-PAGE or immunoblotting.

### Immunoblotting

Protein samples were dissolved in SDS-PAGE loading buffer, boiled for 5 min, and loaded on 10% (v/v) polyacrylamide gel. After running the gel at 100 V for ~1 h, proteins were electrophoretically transferred onto a polyvinylidene fluoride (PVDF) membrane using Semidry Trans-Blot apparatus (Bio-Rad) at 20 V for 20 min. The membrane was blocked in TBST buffer containing 5% (w/v) nonfat milk for 1 h, followed by washing three times with TBST buffer. The membrane was incubated for 1 h with primary antibodies: anti-Flag (GenScript A00187; 1:2000 diluted), anti-6xHis (GenScript A00186; 1:2000 diluted), anti-Histone H3 (GenScript A01502; 1:1000 diluted), anti-PEPC (Rockland 100-4163; 1:500 diluted), and anti-GAPC (Agrisera AS15-2894; 1:2000 diluted). After washing three times with TBST buffer, the membrane was incubated with secondary antibodies (Sigma-Aldrich) from mouse or rabbit conjugated with alkaline phosphatase for 1 h. Proteins were visualized by alkaline phosphatase conjugate substrate (Bio-Rad) according to the manufacturer’s instructions.

### Bimolecular fluorescence complementation (BiFC)

The BiFC vectors were constructed, described, and provided by Walter et al. (2004)^44^. *GAPC1* or *GAPC2* cDNA was cloned into pSPYNE vector (GAPC-YFP^N^), and *NF-YC10* cDNA was cloned into pSPYCE vector (NF-YC10-YFP^C^). Sequence of primers used for cloning is provided in Supplementary Table 1. The constructs were transformed into C58C1 *Agrobacterium tumefaciens* strain and grown to stationary phase. Bacterial cells were collected and resuspended in solution containing 10 mM MES, pH 5.7, 10 mM MgCl_2_, and 150 mg ml^−1^ acetosyringone. Three-week-old *Nicotiana benthamiana* leaves were infiltrated with the mixed bacteria (GAPC-YFP^N^ and NF-YC10-YFP^C^) solutions. YFP fluorescence was examined in tobacco leaves using a Zeiss LSM 510 confocal microscope, with a 488-nm excitation mirror and a 505- to 530-nm filter to record images. GPA1-YFP^N^ and PLDα1-YFP^C^ were used as negative controls for NF-YC10-YFP^C^ and GAPC-YFP^N^, respectively and as a positive control with each other. For quantification of BiFC, confocal images from randomly chosen regions of infiltrated tobacco leaves were obtained with the same settings (magnification, focal plane, area, etc). Background-subtracted fluorescence intensity was measured by ImageJ software (v1.52a).

### Quantitative real-time PCR (qRT-PCR)

Total RNA was extracted from plant tissues using TRIzol Reagent (Life Technologies) per the manufacturer’s instructions. RNA was quantified by Nanodrop 2000 spectrophotometer (Thermo Scientific) and checked for integrity by agarose gel electrophoresis. cDNA was synthesized by SuperScript® III reverse transcriptase (Life Technologies) with 1 μg of RNA and 0.5 μg of oligo(dT)_18_ primers, according to the manufacturer’s instructions. The reaction was at 50 °C for 30 min with preincubation at 65 °C for 5 min and enzyme inactivation at 70 °C for 15 min. The cDNA was amplified with a *Taq* polymerase using gene-specific primers through the following thermal cycling conditions: preincubation at 95 °C for 2 min, 40 cycles of 95 °C for 30 s, 55 °C for 30 s and 68 °C for 1 min, and final extension at 68 °C for 5 min. The PCR progress was monitored by adding SYBR Green dye using StepOnePlus^TM^ Real-Time PCR System (Applied Biosystems), and data were processed by StepOne^TM^ Software (v2.0.2). The gene expression was normalized with *ubiquitin 10* as an internal standard. Sequence of primers used for qRT-PCR is provided in Supplementary Table 1.

### Chlorophyll content measurement

Leaves cut from seedling samples were weighed (*W*), added to 2 mL of 95% (v/v) ethanol in an airtight tube, and agitated at room temperature in dark overnight until leaves turned to white. After brief centrifugation, the ethanol phase was taken and measured with a spectrophotometer at wave lengths of 665 nm (*A*_*665*_), 649 nm (*A*_*649*_), and 470 nm (*A*_*470*_). The amount of chlorophyll a (Ca) was calculated as Ca = 13.95 × *A*_*665*_ - 6.88 × *A*_*649*_; chlorophyll b (Cb) as Cb = 24.96 × *A*_*649*_ - 7.32 × *A*_*665*_; and carotenoids (Cc) as Cc = (1000 × *A*_*470*_ - 2.05 × Ca - 114.8 × Cb)/245. The total content of chlorophyll per fresh weight was calculated as C = 2 × (Ca + Cb + Cc)/*W*.

### Electrolyte leakage and reactive oxygen species measurement

Rosette leaves were excised from 3-week-old plants grown on soil immediately after heat treatment and incubated in 15 mL ultrapure (Milli-Q) water for 1 h at room temperature. Conductivity was measured using Mettler Toledo MC-126 conductivity meter. Samples were then boiled for 15 min and measured again after cooling down to room temperature. Electrolyte leakage was calculated as % conductivity of total ions from the boiled sample. Superoxide anion (O_2_^−^) was measured by staining with nitrotetrazolium blue chloride (NBT). Rosette leaves were cut off from 3-week-old plants after heat treatment and vacuum-infiltrated with 0.1% (w/v) NBT dissolved in 50 mM potassium phosphate and 10 mM sodium azide. The tissues were incubated in the NBT solution for 1 h in the dark with gentle agitation and washed with 95% (v/v) ethanol until completely bleached. Formazan formed was extracted with a mixture of 2 M potassium hydroxide and chloroform (1:1, v/v), dried under gentle stream of nitrogen gas, and dissolved in a mixture of DMSO and 2 M potassium hydroxide (1:1, v/v). Absorbance of the final solution was measured at 700 nm using a spectrophotometer.

### Nuclei isolation

Approximately 1 g of plant tissue was ground with liquid nitrogen in mortar and pestle and mixed with 5 mL of buffer A (10 mM Tris-HCl pH7.6, 0.5 M sucrose, 1 mM spermidine, 4 mM spermine, 10 mM EDTA, and 80 mM KCl). The tissue homogenate was filtered through 4 layers of Miracloth and centrifuged at 3000 × *g* for 5 min. Supernatant was centrifuged at 16000 × *g* for 30 min and used as cytosolic fraction, and pellet (nuclear suspension from the 3000 × *g* centrifugation) was gently resuspended in 1 mL of buffer B (50 mM Tris-HCl pH7.8, 5 mM MgCl_2_, 10 mM β-mercaptoethanol, and 20% (v/v) glycerol). Discontinuous Percoll^TM^ (Amersham Biosciences) gradient was prepared with 2 mL each of 40% (v/v), 60%, and 80% (top to bottom) Percoll^TM^ dissolved in buffer C (25 mM Tris-HCl pH7.5, 0.44 M sucrose, and 10 mM MgCl_2_) on 2 mL of 2 M sucrose cushion. The nuclear suspension was gently loaded on top of the Percoll^TM^ gradient and centrifuged at 4000 × *g* for 30 min. Nuclear layer (light green) just above the 2 M sucrose cushion was carefully taken and washed twice with 1 mL of buffer A at 6000 × *g* for 5 min. The nuclear pellet was gently resuspended in 0.2 mL of buffer B and used for further experiments.

### GAPC activity assay

Catalytic activity of GAPC was determined spectrophotometically by measuring the concomitant reduction of NAD^+^ in a two-step reaction: production of glyceraldehyde-3-phosphate from fructose-1,6-bisphosphate by aldolase followed by 1,3-bisphosphoglycerate from glyceraldehyde-3-phosphate by GAPC. Reaction mixture contained 50 mM triethanolamine-HCl, pH8.5, 4 mM NAD^+^, 1.2 mM fructose-1,6-bisphosphate, 10 mM sodium arsenate, 1 unit of aldolase from rabbit muscle, and 5~20 μg of purified GAPC or total protein extract from Arabidopsis in a final volume of 1 mL. Reaction was initiated by the addition of fructose-1,6-bisphosphate at room temperature for 30 min, then immediately measured for the NADH formation at 340 nm using a spectrophotometer.

### Site-directed mutagenesis and *GAPC*-complemented *gapc1gapc2*

PCR-based lysine mutation of GAPC was carried out using QuikChange Multi Site-Directed Mutagenesis Kit (Agilent Technologies, Santa Clara, CA) according to the manufacturer’s instruction. In brief, *GAPC* cDNA previously cloned in pET-28a(+) vector^7^ was used as a template. Sequence of the mutagenic primers with lysine (AAA or AAG) substituted to alanine (GCA or GCG) is provided in Supplementary Table 1. Mutant strand synthesis was performed by PCR at 95 °C for 1 min followed by 30 cycles of 95 °C 1 min, 55 °C 1 min, and 65 °C 10 min. The PCR product was incubated with restriction enzyme *Dpn* I at 37 °C for 1 h to digest template and used for transformation of XL10-Gold Ultracompetent Cells. The correct mutations were verified by DNA sequencing on both strands. The lysine-mutated *GAPC* cDNA was subcloned into p35S-FAST/eYFP vector for plant expression^7^. In the resulting DNA constructs p35S-FAST/eYFP-GAPCmut and p35S-FAST/eYFP-GAPCmut’, the region containing CaMV-35S promoter and eYFP was replaced with the *GAPC* promoter sequence (−633~−1 from the start codon of *GAPC2*) by cloning at *Eco*RI and *Bam*HI sites of the vector. The DNA constructs pFAST-GAPC::GAPCmut and pFAST-GAPC::GAPCmut’ were then transformed into Agrobacteria, and then into *gapc1gapc2* by floral dipping for the generation of *GAPCmut*- or *GAPCmut’*-complemented *gapc1gapc2*. Transgenic plants were selected on kanamycin and further PCR-confirmed with genomic DNA for the presence of the transgene.

### Protoplast isolation and transfection

Mesophyll protoplasts were isolated from 4-week-old Arabidopsis leaves and transfected with pFAST-35S::NF-YC10-Flag^45^. Briefly, ~0.1 g of leaf strips (~1 mm in width) were vacuum-infiltrated with 5 mL enzyme solution (20 mM MES pH 5.7, 1.5% (w/v) cellulose R10, 0.4% (w/v) macerozyme R10, 0.4 M mannitol, 20 mM KCl, 10 mM CaCl_2_, 0.1% (w/v) BSA) and incubated for 6 h in the dark for cell wall digestion. The protoplast suspension diluted with equal volume of W5 buffer (2 mM MES pH 5.7, 154 mM NaCl, 125 mM CaCl_2_, 5 mM KCl) was filtered through a nylon mesh to remove undigested tissues, then centrifuged at 200 × *g* for 2 min. Pellet was washed with 1 mL W5 solution at 200 × *g* for 2 min and re-suspended in 1 mL MMG buffer (4 mM MES pH 5.7, 0.4 M mannitol, 15 mM MgCl_2_). 100 μL protoplast suspension was mixed with 10 μg DNA and 110 μL PEG solution (20 % (w/v) PEG4000, 0.2 M mannitol, 100 mM CaCl_2_) for 1 h for transfection. The sample diluted with equal volume of W5 buffer was centrifuged at 200 × *g* for 2 min. Pellet was re-suspended in 1 mL WI buffer (4 mM MES pH 5.7, 0.5 M mannitol, 20 mM KCl).

### Chromatin immunoprecipitation (ChIP)

ChIP was performed according to an existing method^46^. For crosslinking, protoplasts washed with PBS buffer (pH 7.4) at 1500 × *g* for 2 min were incubated in 1 mL PBS containing 1% (v/v) formaldehyde for 10 min, then 0.1 M glycine was added for 5 min to quench crosslinking. Protoplasts washed twice with ice-cold PBS were incubated with 1 mL harvest buffer (10 mM DTT, 100 mM Tris-HCl pH 9.4) for 15 min. For chromatin extraction, protoplasts were washed stepwise with ice-cold PBS, nuclei wash buffer with Triton X-100 (0.25 % (v/v) Triton X-100, 10 mM EDTA, 0.5 mM EGTA, 10 mM HEPES pH 6.5), and nuclei wash buffer without Triton X-100 (200 mM NaCl, 1 mM EDTA, 0.5 mM EGTA, 10 mM HEPES pH 6.5), then re-suspended in 300 μL ice-cold nuclei lysis buffer (1% (w/v) SDS, 10 mM EDTA, 50 mM Tris-HCl pH 8.0, 1X protease inhibitor cocktail). DNA was sheared by sonication for 1 min and centrifuged at 10000 × *g* for 5 min. An aliquot of supernatant was reserved as ‘input DNA’. 1 μg of an anti-Flag antibody (GenScript A00187) was added and incubated overnight at 4 °C, then 100 μL swollen protein A agarose (Sigma-Aldrich) was added and incubated for 1 h at 4 °C. The sample was washed stepwise with low salt wash buffer (0.1% (w/v) SDS, 1% (v/v) Triton X-100, 2 mM EDTA, 20 mM Tris-HCl pH 8.0, 150 mM NaCl), high salt wash buffer (low salt buffer with 500 mM NaCl), LiCl wash buffer (0.25 M LiCl, 1% (v/v) NP-40, 1 mM EDTA, 10 mM Tris-HCl pH 8.0, 1% (w/v) sodium deoxycholate), and twice with TE buffer (10 mM Tris-HCl pH 8.0, 1 mM EDTA). 150 μL elution buffer (1% (w/v) SDS, 0.1 M sodium bicarbonate) was added and incubated at 65 °C for 15 min. The sample was centrifuged at 5000 × *g* for 3 min and supernatant was incubated with 0.2 M NaCl at 65 °C for 6 h to reverse crosslinking, then incubated with 1 μg proteinase K at 37 °C for 1 h. DNA was purified using Gel/PCR DNA Fragments Extraction Kit (IBI Scientific) according to manufacturer’s instruction.

### Reporting summary

Further information on research design is available in the Nature Research Reporting Summary linked to this article.

## Supplementary information

Supplementary Information

Reporting Summary

## Data Availability

The data that support the findings of this study are available within the paper and its supplementary information, or are available from the corresponding author upon reasonable request. Source data are provided with this paper. The mass spectrometry proteomics data have been deposited to the ProteomeXchange Consortium via the PRIDE^47^ partner repository with the dataset identifier PXD018945 [10.6019/PXD018945].

## Source data

Source Data
