# Supplementary material for: Nuclear moonlighting of cytosolic glyceraldehyde-3-phosphate dehydrogenase regulates Arabidopsis response to heat stress
Source: Nat Commun. 2020 Jul 10;11:3439. doi: 10.1038/s41467-020-17311-4 (PMC7351759; doi:10.1038/s41467-020-17311-4)
Supplement: Supplementary file 1 — Supplementary Information [file 41467_2020_17311_MOESM1_ESM.pdf]

Supplementary Information

# Nuclear moonlighting of cytosolic glyceraldehyde-3-phosphate dehydrogenase regulates Arabidopsis response to heat stress

Kim et al.

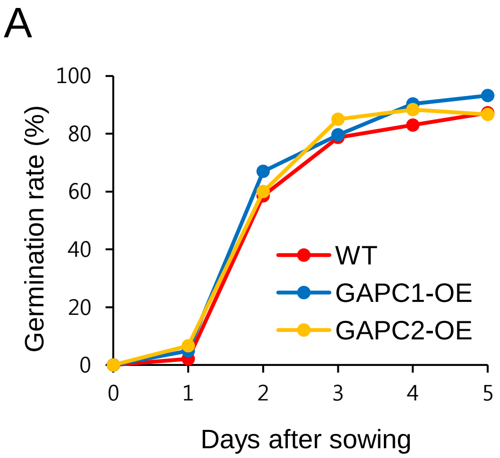


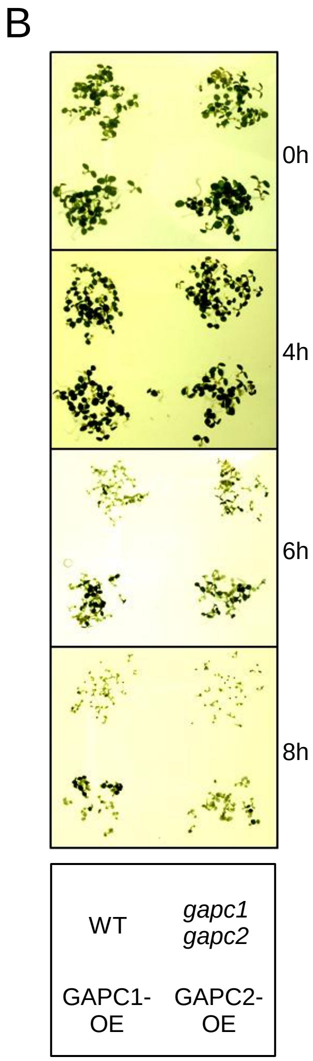


**Supplementary Figure 1. Seed germination and seedling images of *GAPC*-altered Arabidopsis (A)** Germination rate of GAPC-OEs. Desiccated seeds were spread on a wet filter paper. Seeds with visible radicle were scored every day and shown here as % of total seeds. (**B**) Representative images of heat-treated plants. Five-day-old seedlings were treated at 40°C for the indicated times, then placed back to normal temperature condition. Images were taken after 2 days of heat shock. Relative position of plant lines is in the bottom box.

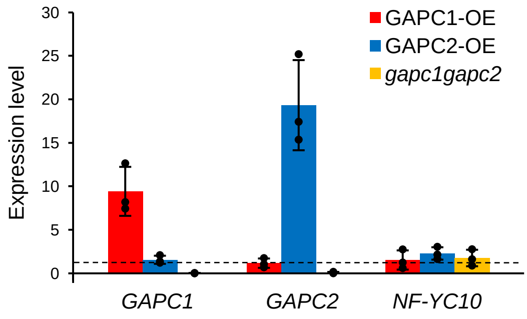


**Supplementary Figure 2. Expression of *GAPC*s and *NF-YC10* in *GAPC*-altered Arabidopsis.** Total RNA was extracted from 5-day-old seedlings of GAPC1-OE, GAPC2-OE, and *gapc1gapc2* treated at 37°C for 5 hours and quantitative RT-PCR was performed with gene-specific primers. Values are average ± S.D. from 3 independent groups of >10 seedlings and shown as fold change to WT (dashed line). Black dots represent individual data points.

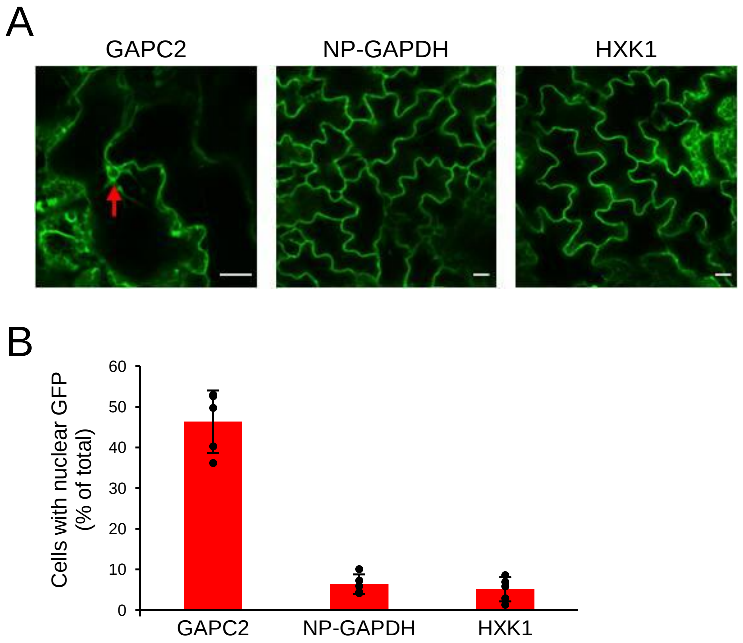


**Supplementary Figure 3. Subcellular localization of other glycolytic enzymes under heat stress.** (**A**) Fluorescence images of Arabidopsis leaf cells. Five-day-old transgenic seedlings overexpressing the indicated GFP-fusion proteins were treated at 40°C for 6 hours and observed under a confocal microscope. GAPC2 was used as a positive control. Arrow indicates the nucleus. Scale bars = 10 μm (**B**) The number of cells with nuclear GFP. Plants were treated and observed as in (A). Cells with clear fluorescence in the nucleus were counted and shown here as % of total cells counted. Values are average ± S.D. from 5 leaves independently treated.

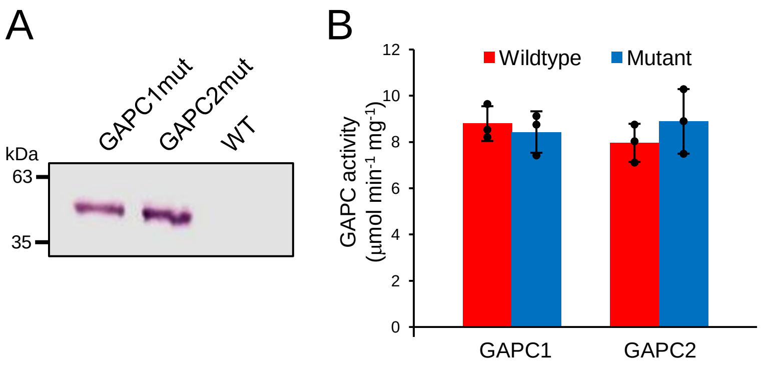


**Supplementary Figure 4. GAPC protein expression in transgenic plants and catalytic activity of purified GAPC** (**A**) Immunoblotting of GAPC. Total proteins were extracted from 5-day-old transgenic seedlings overexpressing GAPC-Flag as indicated on the top. GAPC was probed with an anti-Flag antibody by immunoblotting. (**B**) Catalytic activity. The activity assay was performed by spectrophotometric quantification of NADH formed in the reaction by purified GAPC. Values are average ± S.D. from 3 independent reactions. Mutant, GAPCmut.

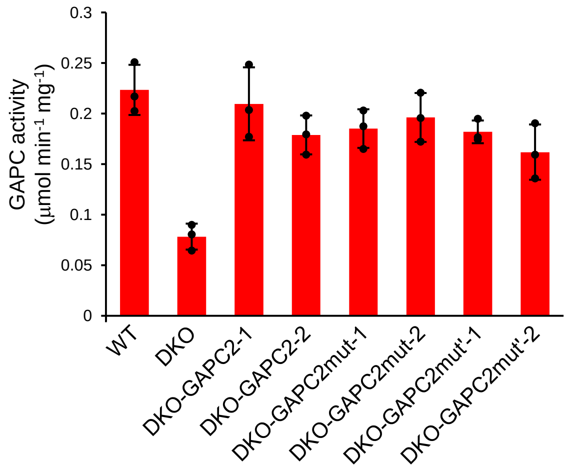


**Supplementary Figure 5. GAPC catalytic activity of *gapc1gapc2* complemented with *GAPC2* variants.** Total proteins were extracted from 10-day-old seedlings. The activity assay was performed by spectrophotometric quantification of NADH formed in the reaction by the protein extracts. Values are average ± S.D. from 3 independent reactions.

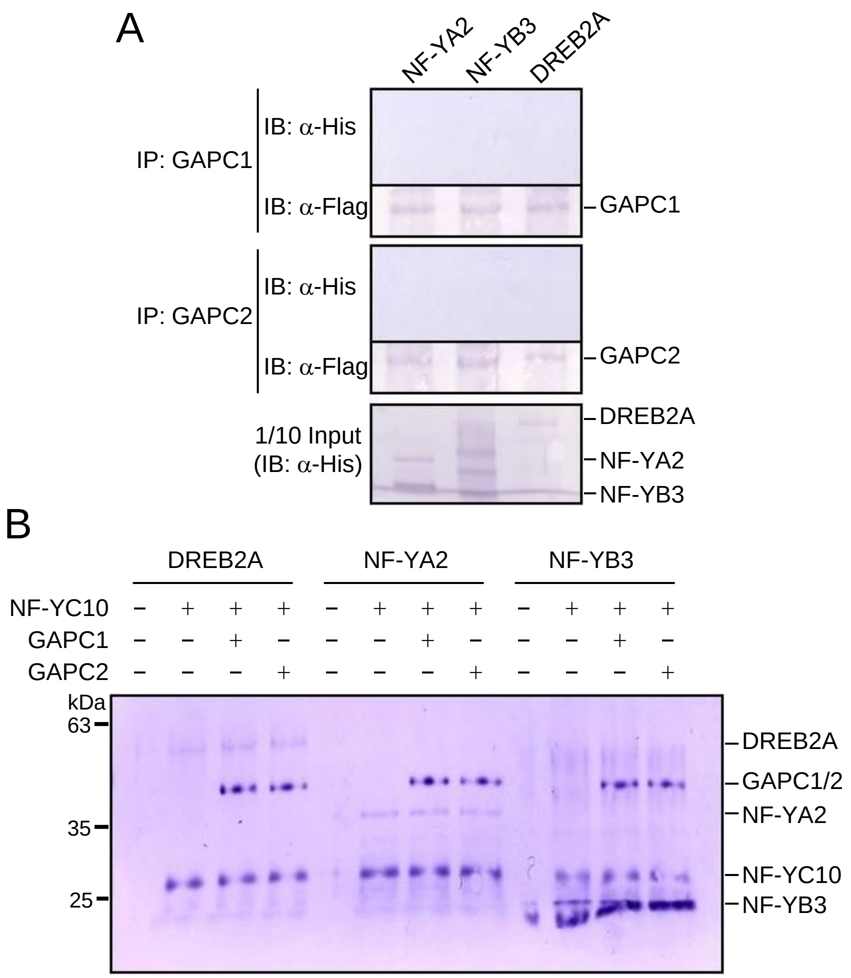


**Supplementary Figure 6. Effect of GAPC on NF-YC10 binding to its target proteins.** (**A**) GAPC interaction with NF-YC10-binding proteins. Purified GAPC-Flag was incubated with NF-YA2-His, NF-YB3-His, or DREB2A-His. GAPC was immunoprecipitated (IP) with an anti-Flag antibody and immunoblotting (IB) was performed with the antibodies indicated on the left. '1/10 Input' denotes protein input with 1/10 of the amount used for the immunoprecipitation. Position of the proteins is indicated on the right. (**B**) Effect of GAPC on NF-YC10 interaction with its binding proteins. Purified NF-YC10-STREP-His was incubated with DREB2A-His, NF-YA2-His, NF-YB3-His, and GAPC1-His or GAPC2-His as indicated above the blot. NF-YC10 was immunoprecipitated with an anti-STREP antibody and immunoblotting was performed with an anti-His antibody. Relative position of the proteins is indicated on the right.

**Supplementary Table 1.** PCR primer and oligonucleotide sequences used in the study

All sequences are listed as 5’→3’. F, forward primer; R, reverse primer

| | | |
|---|---|---|
| For cDNA library construction and cloning | | |
| cDNA library in pDONR221 | F | GTAAAACGACGGCCAG |
| | R | CAGGAAACAGCTATGAC |
| cDNA library in pET-53-DEST | F | AATACGACTCACTATAGGG |
| | R | ATGCTAGTTATTGCTCAGC |
| GAPC1 in pET-28a | F | GCGGGATCCATGGCTGACAAGAAGA |
| | R | GCGAAGCTTTTAGGCCTTTGACATGT |
| GAPC2 in pET-28a | F | GCGGGATCCATGGCTGACAAGAAGATCAGA |
| | R | GCGAAGCTTTTAGGCCTTTGACATGTGAA |
| GAPC1 in p35S-FAST | F | GCGTTAATTAACATGGCTGACAAGAAGATTAGG |
| | R | GCGGTCGACTTAGGCCTTTGACATGTGGACGAT |
| GAPC2 in p35S-FAST | F | GCGTTAATTAACATGGCTGACAAGAAGATCAGA |
| | R | GCGGTCGACTTAGGCCTTTGACATGTGA |
| GAPC1 in pSPYNE | F | GCGTCTAGAATGGCTGACAAGAAGAT |
| | R | CGCGGTACCGGCCTTTGACATGTGGA |
| GAPC2 in pSPYNE | F | GCGTCTAGAATGGCTGACAAGAAGATCAGA |
| | R | CGCGGTACCGGCCTTTGACATGTGAACG |
| NF-YC10 in pET-53-DEST | F | GGGGACAAGTTTGTACAAAAAAGCAGGCTTCATGGTGTCGTCAAAGAAACCCAAGGAG |
| | R | GGGGACCACTTTGTACAAGAAAGCTGGGTTGCCTGCATCTGTCATGCCTCTTTCC |
| NF-YC10 in p35S-FAST | F | GCGTCTAGAATGGTGTCGTCAAAGAAACC |
| | R | CGCGGTACCGCCTGCATCTGTCATGCCTC |
| NF-YC10 in pSPYCE | F | CGCGGATCCATGGTGTCGTCAAAGAAACC |
| | R | CCGCTCGAGGCCTGCATCTGTCATGCCTC |
| NF-YA2 in pET-28a | F | GGCCAAGCTTGCATGGCTATGCAAACTGTG |
| | R | GGCCCTCGAGTCAGGTTTTGAAATTGCAG |
| NF-YB3 in pET-28a | F | GGCCAAGCTTGCATGGCGGATTCGGACAAC |
| | R | GGCCCTCGAGTTAAGAAAAATGATGGGAA |
| DREB2A in pET-28a | F | CGCGGATCCATGGCAGTTTATGATCAGAG |
| | R | CCCAAGCTTTTAGTTCTCCAGATCCAAG |
| GAPC promoter | F | CCGGAATTCCGAGTTTTTGATAGGGACTTTTGC |
| | R | CGCGGATCCTGTAGAATCGAAAACGAGAGTTAG |
| For quantitative real time-PCR | | |
| HsfA2 | F | TGGGATTCTCATAAGTTCTCAACA |
| | R | TGGATCAATCTTTCTGAATCCAT |
| HsfA3 | F | TTCGCTAACGAGGCTTTCC |
| | R | CCTCAGTAGGTGACCCTT |
| HsfA7A | F | GCTCTAGAATGATGAACCCGTTTCTCCCG |
| | R | TCCCCCGGGTTAGGAGGTGGAAGCCAAAC |
| HsfA7B | F | AGCAGATTTTCGAGCAGAAGAGA |
| | R | TGCTCCACCTCTTCCATTTTGAT |
| Hsp17.6A-CI | F | CTGGGTCTTGACTTTGTGTGTG |
| | R | TGTCACACAAGTTACTAGCTTCCA |
| At1g75860 | F | GTTGGCGTTCAAAACTTGGTCG |
| | R | CTCTAGTGAGTCAGACTCATCTG |
| At4g36010 | F | CTTGTGGCGGAGCTGATTAC |
| | R | CCTTCGTTGCACTCTTCACA |
| Hsp70b | F | ATGGGATCTTAAATGTGTCG |

| | | |
|---|---|---|
| | R | CCTGAGCTACTGAAATCACC |
| Hsp15.7-CI(r) | F | TGATCAACTTTCACATTCTCC |
| | R | TGATCAACTTTCACATTCTCC |
| LFG4 | F | TAACCGTCTACACCTTTTGG |
| | R | CAGCTCTGAAAATGGTAAGG |
| EGY3 | F | GAGAAGACGAGAACTGATCG |
| | R | CCCACTCATAAGAGCTATGG |
| CLPB3 | F | ATTGTGCAAGGAGATGTACC |
| | R | CTATCTGAGATGCGAACTCC |
| Hsp15.4-CI(r) | F | AAGAGAATCATGTTCGTTGG |
| | R | CTTTCAGGAACATCAGAAGG |
| FBS1 | F | GACAAAACTACTTCGGTTGC |
| | R | CAAGTTGTTTGATTCAGTGG |
| SAP10 | F | TAGCAAACACTTATCGAACG |
| | R | ATGGTAATGCTTCTGTTTCG |
| DREB2C | F | GCCTAAGTTTGTGATTCTGC |
| | R | CTTGCCAAGTCAATAAAACC |
| At1g75960 | F | CTAAAGGAGTGGTTCATTGC |
| | R | CGATGAAACCTAGAGACTCG |
| ACS7 | F | CGTCTACAGTCTCTCCAAGG |
| | R | TTAAGATCACATCCCAAAGC |
| For site-directed mutagenesis | | |
| GAPC-K121A | GAAGGGTGGTGCTGCAAAGGTTGTCATC | |
| GAPC-K130A | CTGCCCCAAGCGCAGATGCGCCCATGTTC | |
| GAPC-K219A | CACTGGTGCCGCCGCGGCTGTTGGGAAAG | |
| GAPC-K223A | CAAGGCTGTTGGGGCAGTGTTGCCATC | |
| GAPC-K231A | CATCCCTCAATGGAGCATTGACCGGAATGTC | |
| GAPC-K255A | CGTTAGACTTGAGGCAGCTGCAACATACG | |
| For chromatin immunoprecipitation | | |
| HsfA2 | F | AGAGAAAAATTGTGCAGCAGGT |
| | R | CGCCAGAAAAAGCCTACTAAAA |
| HsfA3 | F | GAGAGCTAAGTGAAGCTGCAAGGA |
| | R | TCGTCATCATGTTCCATTGATT |
| At1g75860 | F | CGGACCGAGCCAGTAGTCGTC |
| | R | GGGGGAGAAGATAGCTAAGCGCG |
