# Supplementary material for: Nuclear moonlighting of cytosolic glyceraldehyde-3-phosphate dehydrogenase regulates Arabidopsis response to heat stress
Source: Nat Commun. 2020 Jul 10;11:3439. doi: 10.1038/s41467-020-17311-4 (PMC7351759; doi:10.1038/s41467-020-17311-4)
Supplement: Supplementary file 2 — Reporting Summary [file 41467_2020_17311_MOESM2_ESM.pdf]

nature research

Corresponding author(s): Xuemin Wang

Last updated by author(s): Jun 15, 2020

Reporting Summary

Nature Research wishes to improve the reproducibility of the work that we publish. This form provides structure for consistency and transparency in reporting. For further information on Nature Research policies, see our Editorial Policies and the Editorial Policy Checklist.

Statistics

For all statistical analyses, confirm that the following items are present in the figure legend, table legend, main text, or Methods section.

n/a	Confirmed	
☐	☒	The exact sample size (*n*) for each experimental group/condition, given as a discrete number and unit of measurement
☐	☒	A statement on whether measurements were taken from distinct samples or whether the same sample was measured repeatedly
☐	☒	The statistical test(s) used AND whether they are one- or two-sided<br>*Only common tests should be described solely by name; describe more complex techniques in the Methods section.*
☒	☐	A description of all covariates tested
☒	☐	A description of any assumptions or corrections, such as tests of normality and adjustment for multiple comparisons
☐	☒	A full description of the statistical parameters including central tendency (e.g. means) or other basic estimates (e.g. regression coefficient) AND variation (e.g. standard deviation) or associated estimates of uncertainty (e.g. confidence intervals)
☒	☐	For null hypothesis testing, the test statistic (e.g. *F*, *t*, *r*) with confidence intervals, effect sizes, degrees of freedom and *P* value noted<br>*Give P values as exact values whenever suitable.*
☒	☐	For Bayesian analysis, information on the choice of priors and Markov chain Monte Carlo settings
☒	☐	For hierarchical and complex designs, identification of the appropriate level for tests and full reporting of outcomes
☒	☐	Estimates of effect sizes (e.g. Cohen's *d*, Pearson's *r*), indicating how they were calculated

Our web collection on statistics for biologists contains articles on many of the points above.

Software and code

Policy information about availability of computer code

Data collection	StepOne Software v2.0.2 for gene expression data collection; Zeiss LSM 510 for microscope image collection
Data analysis	ImageJ 1.48v for image analysis; StepOne Software v2.0.2 for relative gene expression analysis; Microsoft Excel 2016 for plotting graphs and for statistical analysis

For manuscripts utilizing custom algorithms or software that are central to the research but not yet described in published literature, software must be made available to editors and reviewers. We strongly encourage code deposition in a community repository (e.g. GitHub). See the Nature Research guidelines for submitting code & software for further information.

Data

Policy information about availability of data

All manuscripts must include a data availability statement. This statement should provide the following information, where applicable:

- Accession codes, unique identifiers, or web links for publicly available datasets
- A list of figures that have associated raw data
- A description of any restrictions on data availability

The data that support the findings of this study are available within the paper and its supplementary information, or are available from the corresponding author upon reasonable request. The source data for Figures 1AB, 2AB and D, 3A-F, 4A-C, 5A and C-G, 6B and D-G, 7B and D-F, 8A-E, 9A-C and Supplementary Figures 1A, 2, 3B, 4AB, 5, and 6AB are provided as a Source Data File. The mass spectrometry proteomics data have been deposited to the ProteomeXchange Consortium via the PRIDE47 partner repository with the dataset identifier PXD018945 [http://dx.doi.org/10.6019/PXD018945].

Field-specific reporting

Please select the one below that is the best fit for your research. If you are not sure, read the appropriate sections before making your selection.

- [x] Life sciences
- [ ] Behavioural & social sciences
- [ ] Ecological, evolutionary & environmental sciences

For a reference copy of the document with all sections, see nature.com/documents/nr-reporting-summary-flat.pdf

Life sciences study design

All studies must disclose on these points even when the disclosure is negative.

Sample size	Samples sizes were chosen on the basis of preliminary experiments (and previous publications) to provide sufficient power for statistical comparisons. No sample-size calculation was performed.
Data exclusions	No data were excluded from this study.
Replication	At least three replications were used for each experiment.
Randomization	Plant materials were randomly picked up for our data collection and analyses.
Blinding	Complete blinding for data collection was not possible and necessary. However, the individual plants constituting biological replicates were chosen as randomly as possible to perform partially-blinded experiments.

Reporting for specific materials, systems and methods

We require information from authors about some types of materials, experimental systems and methods used in many studies. Here, indicate whether each material, system or method listed is relevant to your study. If you are not sure if a list item applies to your research, read the appropriate section before selecting a response.

Materials & experimental systems

n/a	Involved in the study	
☐	☒	Antibodies
☒	☐	Eukaryotic cell lines
☒	☐	Palaeontology and archaeology
☒	☐	Animals and other organisms
☒	☐	Human research participants
☒	☐	Clinical data
☒	☐	Dual use research of concern

Methods

n/a	Involved in the study	
☒	☐	ChIP-seq
☒	☐	Flow cytometry
☒	☐	MRI-based neuroimaging

Antibodies

Antibodies used	Monoclonal anti-6xHis antibody (GenScript #A00186); Monoclonal anti-FLAG antibody (GenScript #A00187); Polyclonal anti-histone H3 antibody (GenScript #A01502); Polyclonal anti-STREP antibody (GenScript #A00626); Polyclonal anti-PEPC antibody (Rockland #100-4163); Polyclonal anti-GAPC antibody (Agrisera #AS15-2894); Anti-mouse IgG-alkaline phosphatase antibody (Sigma-Aldrich #A1293); Anti-rabbit IgG-alkaline phosphatase antibody (Sigma-Aldrich #A7539)
Validation	Information of the primary antibodies are available in the following manufacturer's websites:<br>Monoclonal anti-6xHis antibody: https://www.genscript.com/antibody/A00186_Q-THE_sup_TM_sup_His_Tag_Antibody_mAb_Mouse.html<br>Monoclonal anti-FLAG antibody: https://www.genscript.com/antibody/A00187_Q-_THE_sup_TM_sup_DYKDDDDK_Tag_Antibody_mAb_Mouse.html<br>Polyclonal anti-histone H3 antibody: https://www.genscript.com/antibody/A01502_40-Histone_H3_antibody_pAb_Rabbit.html<br>Polyclonal anti-STREP antibody: https://www.genscript.com/antibody/A00626_40-NWSHPQFEK_Antibody_pAb_Rabbit.html<br>Polyclonal anti-PEPC antibody: https://rockland-inc.com/Product.aspx?id=42104<br>Polyclonal anti-GAPC antibody: https://www.agrisera.com/en/artiklar/gapc1-2-glyceraldehyde-3-phosphate-dehydrogenase.html
